# Biomimetic electrodynamic nanoparticles comprising ginger-derived extracellular vesicles for synergistic anti-infective therapy

**DOI:** 10.1038/s41467-022-34883-5

**Published:** 2022-11-22

**Authors:** Zhuangzhuang Qiao, Kai Zhang, Jin Liu, Daojian Cheng, Bingran Yu, Nana Zhao, Fu-Jian Xu

**Affiliations:** 1 State Key Laboratory of Chemical Resource Engineering, Beijing University of Chemical Technology, Beijing, 100029 China; 2 Key Laboratory of Biomedical Materials of Natural Macromolecules (Beijing University of Chemical Technology), Ministry of Education, Beijing, 100029 China; 3 Beijing Laboratory of Biomedical Materials, Beijing University of Chemical Technology, Beijing, 100029 China; 4 State Key Laboratory of Organic-Inorganic Composites, Beijing University of Chemical Technology, Beijing, 100029 China

**Keywords:** Drug delivery, Biomaterials, Biomedical materials, Nanobiotechnology

## Abstract

Nanotechnology enlightens promising antibacterial strategies while the complex in vivo infection environment poses a great challenge to the rational design of nanoplatforms for safe and effective anti-infective therapy. Herein, a biomimetic nanoplatform (EV-Pd-Pt) integrating electrodynamic Pd-Pt nanosheets and natural ginger-derived extracellular vesicles (EVs) is proposed. The introduction of ginger-derived EVs greatly endows EV-Pd-Pt with prolonged blood circulation without immune clearance, as well as accumulation at infection sites. More interestingly, EV-Pd-Pt can enter the interior of bacteria in an EV lipid-dependent manner. At the same time, reactive oxygen species are sustainably generated in situ to overcome the limitations of their short lifetime and diffusion distance. Notably, EV-Pd-Pt nanoparticle-mediated electrodynamic and photothermal therapy exhibit synergistic effects. Furthermore, the desirable biocompatibility and biosafety of the proposed nanoplatform guarantee the feasibility of in vivo applications. This proof-of-concept work holds significant promise for developing biomimetic nanoparticles by exploiting their intrinsic properties for synergistic anti-infective therapy.

## Abstract (web summary)

Antibacterial materials often suffer from issues around safety and application in complex in vivo environments. Here the authors report on the conjugation of electro-driven catalytic Pd-Pt nanosheets on ginger-derived extracellular vesicles for bacterial uptake and synergistic antibacterial therapy.

## Introduction

Pathogenic bacterial infection seriously threatens human health and life, which has received widespread attention^1,2^. Due to the antibiotic-resistance crisis, there is an urgent need to develop alternative strategies to combat bacterial infections while a variety of nanomaterials have been proposed to construct antibacterial platforms^3–5^. Compared with traditional approaches, the introduction of nanotechnology into antibacterial applications has achieved satisfactory therapeutic results, including photothermal therapy (PTT)^6–8^ and diverse dynamic therapies^9,10^. In particular, reactive oxygen species (ROS)-mediated dynamic treatment strategies demonstrate great potential in anti-infective therapy which could cause irreversible damage to essential lipids, DNA, and proteins, such as photodynamic therapy (PDT)^11,12^, chemodynamic therapy (CDT)^6,8^, sonodynamic therapy (SDT)^13^, and electrodynamic therapy (EDT)^14^. Generally speaking, the generation efficiency of ROS is either dependent on the external triggers (e.g., light, ultrasound, and electric field) or internal stimuli such as endogenous hydrogen dioxide (H_2_O_2_). However, the hypoxia and insufficient H_2_O_2_ content in the infectious microenvironment greatly limits the therapeutic effectiveness of the oxygen-dependent PDT, SDT, and H_2_O_2_-dependent CDT^15–17^. As an emerging dynamic therapeutic strategy, EDT stimulated by electric field can produce hydroxyl radicals (·OH) in a sustainable and microenvironment-independent manner^9,18–21^, showing distinct advantages in bacterial infection and clinical translation. In most cases, noble metal Pt nanoparticles are employed as nano-electrosensitizers for EDT-mediated tumor treatment^18^. To further improve therapeutic efficacy, the combination of EDT with chemotherapy^20,21^, CDT^22^, PDT^14^, starvation therapy^19^, and immunotherapy^23^ has been proposed. On the other hand, enhanced EDT was achieved by the rational design of nano-electrosensitizers. For example, Pt-Cu alloy nanoparticles was found to promote ROS production through increasing the intracellular concentration of chloride ion^24^. The introduction of Pd in the composite Pt-Pd nanoparticles could realize synergistic electrocatalytic effect with Pt^23^. However, the underlying functional mechanisms by which the tailored nano-electrosensitizers improve catalytic activity remain to be further investigated. In addition, the application of EDT was limited to tumor treatment^18–24^ and antibacterial wound dressing^14^, while there is a great need to explore more disease models. Meanwhile, strong interactions between nano-electrosensitizers and bacteria are required to overcome the short lifetime and diffusion distance of ·OH to achieve improved bactericidal effect^25,26^. Therefore, the development of orchestrated nanoplatforms to achieve efficient EDT in complex in vivo infection environments still remains a great challenge.

As nano-sized membrane vesicles, extracellular vesicles (EVs) secreted by cells have shown great efficiency in the construction of biomimetic nanoplatforms^27,28^. In addition to excellent biocompatibility and blood circulation stability, the natural cellular uptake and inherent targeting properties of EVs originating from the cell membrane proteins on the surface make them ideal candidates for delivery systems^29,30^. Compared with EVs derived from mammalian cells, plant-derived edible EVs with low immunological risk and intrinsic therapeutic activity are green, sustainable, renewable, and mass-producible^31–34^. Ginseng-derived EVs are efficiently taken up by bone marrow derived mesenchymal stem cells to stimulate neural differentiation, while grapefruit EVs are able to deliver therapeutic agents or nanoparticles with enhanced cellular internalization for tumor treatment^29,32,33^. More interestingly, ginger-derived EVs are found to be selectively internalized by gut bacteria (*Lactobacillus rhamnosus*) or periodontal pathogen (*Porphyromonas gingivalis*) in an EV lipid-dependent manner to shape the gut microbiota or inhibit pathogenicity^35,36^. The unique property of EVs that can be taken up by bacteria inspired us to propose a more efficient antibacterial nanoplatform through integrating EVs and superior nano-electrosensitizers. In this context, it is expected that both enhanced accumulation of nanoparticles at infection sites without immune clearance and strong interaction between ·OH and bacteria can be achieved for improved EDT and bactericidal effect.

**Fig. 1:**
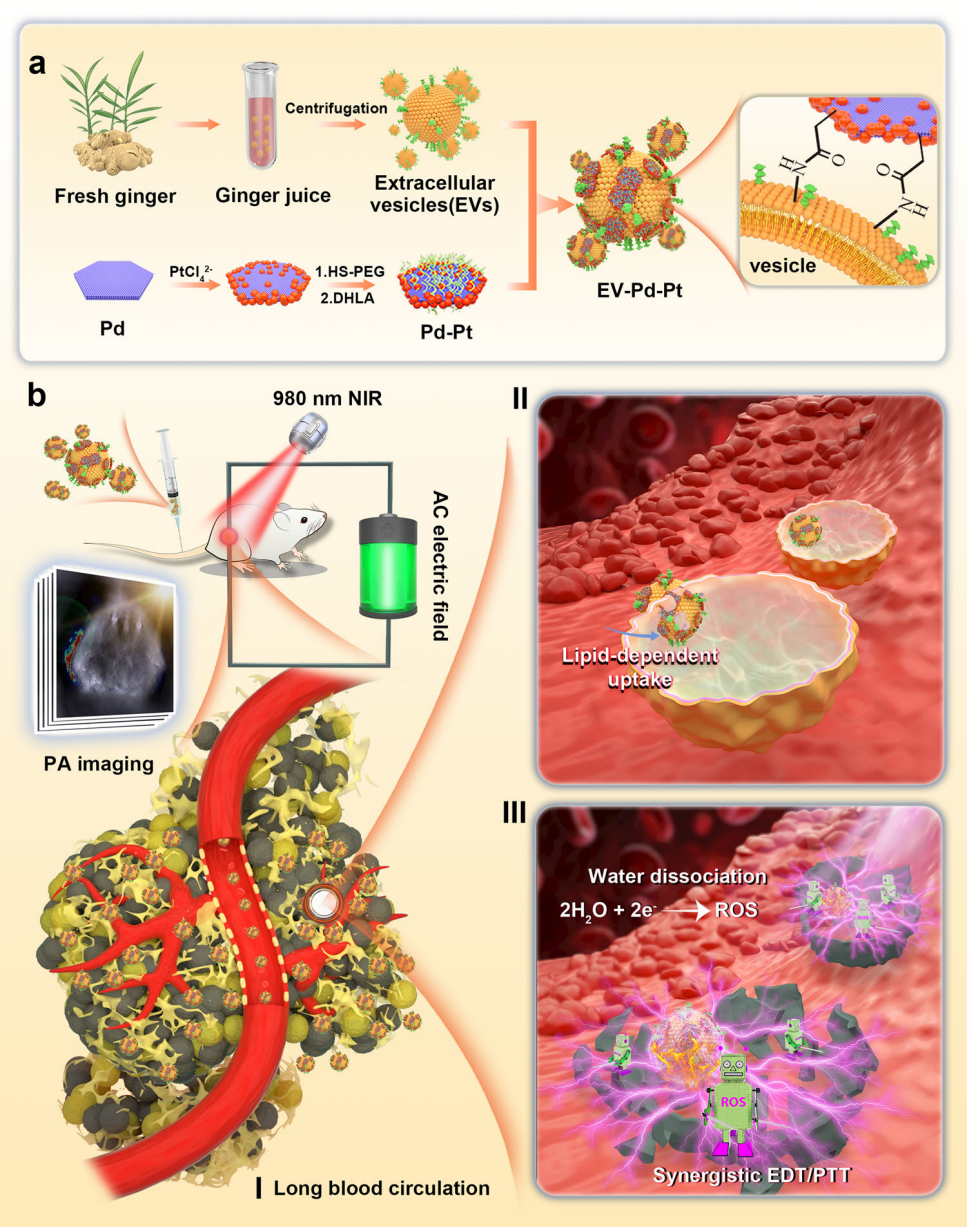
Schematic of the preparation of the EV-Pd-Pt nanoplatform and its antibacterial application. **a** Schematic illustration of the preparation of EV-Pd-Pt nanoparticles by conjugating carboxylic group-functionalized Pd-Pt nanoparticles onto the surface of ginger-derived EVs with abundant amino groups. **b** PA imaging-guided synergistic EDT/PTT of bacterial infection in vivo can be achieved by applying electric field and NIR light irradiation after intravenous injection of EV-Pd-Pt. Long blood circulation, efficient accumulation at infection site, and lipid-dependent cellular uptake are hypothesized to favor antibacterial efficacy.

Herein, we demonstrate a rational design of a biomimetic nanoplatform (EV-Pd-Pt) by combining ginger-derived EVs and Pd-Pt nanosheets for synergistic bacteria eradication (Fig. 1). Pd-Pt nanosheets were conjugated onto the surface of ginger-derived EVs via the amide reaction between carboxylic group-functionalized Pd-Pt and abundant amino groups on the surface of EVs to construct the nanoplatform. The electro-driven catalytic activity of Pd-Pt nano-electrosensitizers was supposed to be enhanced compared with Pt nanoparticles, which was verified by both experimental data and computational simulation. The excellent biocompatibility and long blood circulation time introduced by EVs guarantee the accumulation of EV-Pd-Pt at the infection sites without immune elimination. More importantly, EV-Pd-Pt was hypothesized to enter the interior of bacterial cells in an EV lipid-dependent manner and generate ROS in situ, resulting in strong interaction with bacteria and efficient eradication of bacterial infection by high-performance EDT. In addition, taking advantage of the photothermal property of Pd-Pt, photoacoustic (PA) imaging-guided synergistic EDT/PTT could be achieved since the catalytic activity of Pd-Pt could be further enhanced by heat induced by near infrared (NIR) light irradiation. Furthermore, the bactericidal effect in vivo was explored by a mouse model of subcutaneous abscesses infected with *Staphylococcus aureus* (*S. aureus*).

## Results

### Preparation and characterization of EV-Pd-Pt nanoparticles

**Fig. 2:**
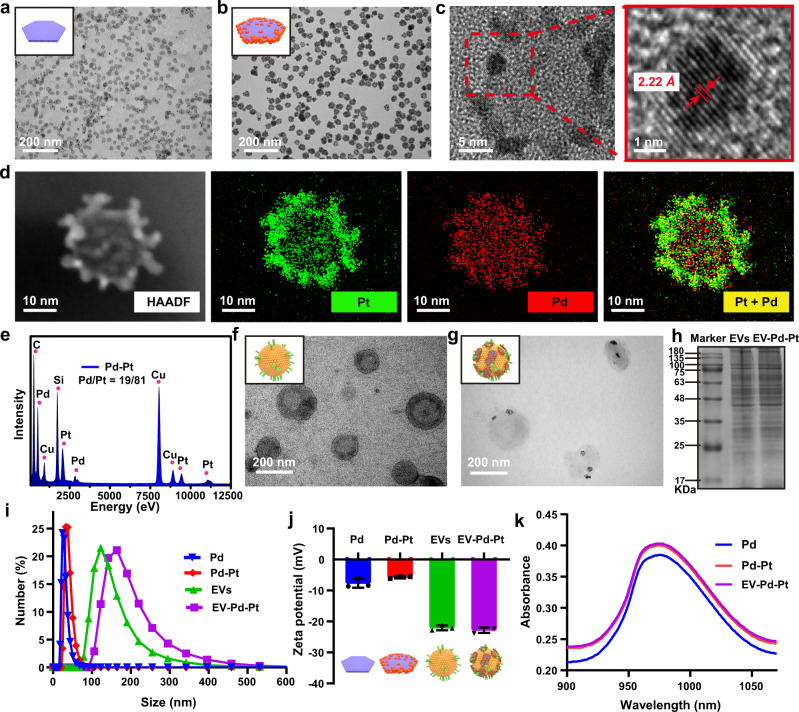
Characterization of EV-Pd-Pt nanoparticles. TEM images of Pd (**a**) and Pd-Pt (**b**) nanosheets. **c** Enlarged and high-resolution TEM images of Pd-Pt nanosheets. **d** Scanning TEM elemental mapping of Pd-Pt nanosheets. **e** EDS of Pd-Pt nanosheets. TEM images of EVs extracted from the ginger juice (**f**) and EV-Pd-Pt (**g**). **h** SDS-PAGE protein analysis of EVs and EV-Pd-Pt. **a**, **b**, **d**, **f**–**h**, Experiments were repeated three times independently with similar results. Size distribution (**i**) and zeta potential (**j**) of Pd, Pd-Pt, EVs, and EV-Pd-Pt from DLS analysis. Data are presented as mean values ± SD (*n* = 3 independent samples). **k** Absorption spectra of Pd, Pd-Pt, and EV-Pd-Pt. Source data are provided as a Source Data file.

The Pd-Pt nano-electrosensitizers were prepared through a facile seed-mediated growth strategy employing Pd nanosheets as seeds and polyvinylpyrrolidone as a stabilizer^37,38^. As shown in the transmission electron microscopy (TEM) images (Fig. 2a, b and Supplementary Fig. 1), well-dispersed Pd nanosheets with a uniform size of ~22 nm were synthesized while the size of Pd-Pt nanosheets was ~30 nm. The Pd-Pt nanosheets were observed to display surface roughness with small Pt nanoparticles deposited on Pd nanosheets (Fig. 2c). The lattice spacing of ~2.22 Å corresponding to the (111) plane of Pt confirms the crystal structure of Pt nanoparticles. The elemental mapping images of Pd-Pt nanosheets (Fig. 2d) suggest the preferential distribution of Pt element on the edges of Pd nanosheets, verifying the successful preparation of the Pd-Pt heterostructures. The mass ratio of Pd:Pt was determined to be ~1:4 by energy-dispersive X-ray spectroscopy (EDS, Fig. 2e). Ginger-derived EVs were then isolated and purified by differential centrifugation and sucrose gradient centrifugation methods. As shown in the TEM image (Fig. 2f), the extracted vesicles from ginger exhibit an obvious saucer-like shape of typical EVs. Thereafter, Pd-Pt nanosheets were functionalized with carboxylic groups and patched onto the surface of ginger-derived EVs through amidation reaction to generate EV-Pd-Pt (Fig. 2g). As exhibited in Supplementary Fig. 2, the elemental mapping reveals the co-localization of Pd and Pt elements on EVs, demonstrating the formation of EV-Pd-Pt. The high-resolution X-ray photoelectron spectroscopy (XPS) spectra of N1s show the relatively lower peak intensity of –NH_2_ and higher intensity of N-C in EV-Pd-Pt compared with EVs (Supplementary Fig. 3), confirming the successful conjugation of Pd-Pt nanosheets onto EVs via amide bonds. The protein components in EV-Pd-Pt analyzed by sodium dodecyl sulfate-polyacrylamide gel electrophoresis (SDS-PAGE) suggest that the primary membrane proteins from EVs were mostly retained in biomimetic EV-Pd-Pt (Fig. 2h). The hydrodynamic size of EV-Pd-Pt obtained by dynamic light scattering (DLS) was found to increase from ~150 of EVs to ~190 nm (Fig. 2i), indicating the successful fabrication of EV-Pd-Pt. The weight ratio of Pd-Pt in EV-Pd-Pt was calculated to be ~20% by thermogravimetric analysis. As shown in Fig. 2j, the zeta potential of EV-Pd-Pt decreased from ~−5.7 mV of Pd-Pt to ~−22.8 mV, which is similar to the zeta potential of pure EVs (~−22.1 mV). Compared with Pd-Pt nanosheets, the larger repulsive force between EV-Pd-Pt nanoparticles guarantees their enhanced stability in blood. Additionally, the corresponding absorption spectra indicate that EV-Pd-Pt exhibits the characteristic absorption peak of Pd-Pt at 980 nm (Fig. 2k).

### Electrodynamic and photothermal properties of Pd-Pt nanosheets

**Fig. 3:**
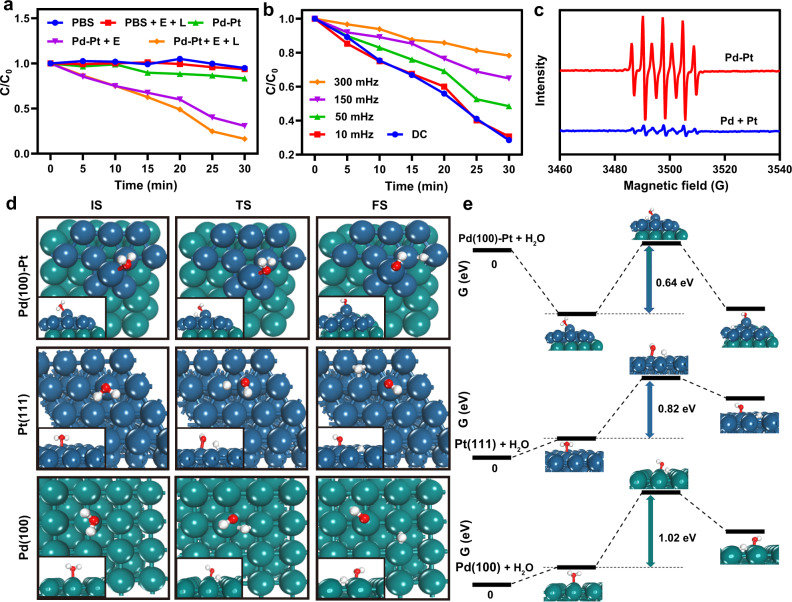
Electro-driven catalytic performance of Pd-Pt nanosheets. **a** Degradation behaviors of MB under different treatments. **b** Degradation rates of MB under AC electric field with different frequencies or DC field in the presence of Pd-Pt nanosheets. **c** ESR spectra of the system with Pd-Pt nanosheets and the simple mixture of Pd and Pt nanoparticles, respectively employing DMPO as the spin-trapping agent. Theoretical calculation schematics of initial (IS), transition (TS), and final (FS) states (**d**) and energy profiles (**e**) of water dissociation reactions on the Pd(100)-Pt, Pt(111), and Pd(100) surfaces. White, red, blue, and green spheres represent H, O, Pt, and Pd atoms, respectively. Source data are provided as a Source Data file.

The electro-driven catalytic activity of Pd-Pt nanosheets was systematically investigated using methylene blue (MB) as the ROS indicator in a double salt bridge system (Supplementary Fig. 4). As shown in Fig. 3a, the characteristic absorption of MB at 664 nm only decreased negligibly and slightly in the PBS (as control), alternating current (AC) power supply with 980 nm NIR laser irradiation (E + L), and Pd-Pt nanosheet groups. In contrast, obvious MB degradation was observed in the presence of Pd-Pt nanosheets under square-wave AC electric field (E), suggesting the efficient generation of ·OH. After the introduction of 980 nm NIR laser irradiation (L), the MB degradation rate was further accelerated, implying that the NIR laser-induced heat could enhance the generation efficiency of ·OH. In addition, the square-wave frequency-dependent MB degradation behaviors were found (Fig. 3b). Compared with the DC field group, the degradation rate of MB gradually slowed down with the increase of the square-wave frequency of AC field. Therefore, the square-wave AC field with an optimal frequency of 10 mHz was selected for the subsequent EDT studies. The ·OH generation under the AC field was also verified by the electron spin resonance (ESR) spectra (Fig. 3c). The oxidation of 2,2,6,6-tetramethylpiperidine (DMPO) by two ·OH radicals produced characteristic peaks with intensity of 1:2:1:2:1:2:1^39^. Interestingly, the ·OH signal triggered by Pd-Pt nanosheets was much stronger than that triggered by a simple mixture of Pd nanosheets and Pt nanoparticles with equivalent Pd and Pt contents. Taken together, Pd-Pt nanosheets exhibit excellent catalytic activity under square-wave AC field to produce highly cytotoxic ·OH, demonstrating their great potential in EDT-mediated antibacterial therapy. Furthermore, EV-Pd-Pt showed a MB degradation rate consistent with Pd-Pt nanosheets at the same Pd-Pt concentration (Supplementary Fig. 5), indicating the comparable electrodynamic performance of EV-Pd-Pt and Pd-Pt.

Notably, to explore the mechanism of electro-driven catalytic activity of Pd-Pt nanosheets, density functional theory (DFT) calculations were performed to simulate the dissociation reaction of H_2_O molecules. In our model, atoms of Pt(111) were arranged on the Pd(100) substrate to represent interfacial interaction of Pd(100)-Pt hetero-nanoparticles. Firstly, the adsorption energies of H_2_O on Pt(111), Pd(100), and Pd(100)-Pt surfaces were calculated to be 0.28, 0.26, and −0.58 eV, respectively. Compared with the Pt(111) and Pd(100) surfaces, H_2_O would be more favorably adsorbed on the surface of Pd(100)-Pt, which is beneficial to the subsequent water disassociation. The schematics and energy profiles of water dissociation on active sites of Pt(111), Pd(100) and Pd(100)-Pt surfaces are presented in Fig. 3d, e. The energy barrier for water molecule dissociation on the Pd(100)-Pt surface was calculated to be ~0.64 eV, which is much lower than that on other surfaces. These results indicate that H_2_O is easier to dissociate into hydroxyl radicals on the surface of Pd-Pt than Pt or Pd nanoparticles, which is consistent with the MB degradation results.

Furthermore, the absorption of Pd-Pt nanosheets in the desirable NIR region (Fig. 2k) inspired us to further evaluate their photothermal effects. The temperature increases of the solution showed a concentration- and time-dependent pattern after exposure to a 980 nm NIR laser (Supplementary Fig. 6). In contrast, negligible temperature change was observed for the pure water, illustrating the excellent photothermal performance of Pd-Pt. Meanwhile, the color change in photothermal images and temperature increase of EV-Pd-Pt under NIR laser irradiation were observed to be almost identical to those of Pd-Pt (Supplementary Fig. 7), indicating the negligible effect of EVs on the photothermal property of EV-Pd-Pt. The favorable electrodynamic and photothermal properties of Pd-Pt nanosheets and EV-Pd-Pt endow them with great potential for antibacterial applications.

### Uptake of EV-Pd-Pt nanoparticles by bacteria

**Fig. 4:**
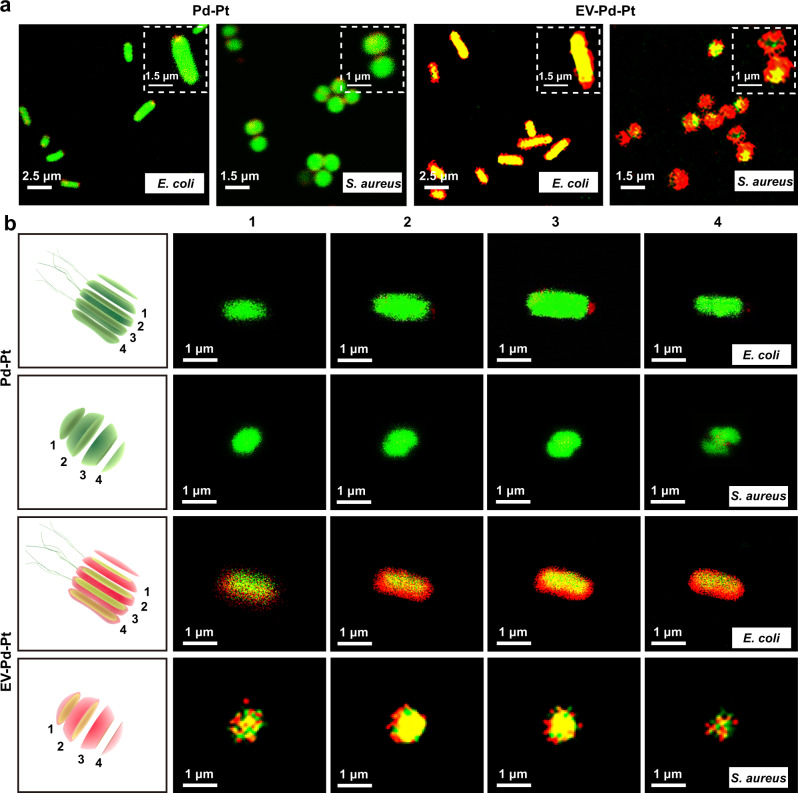
Interaction of EV-Pd-Pt and Pd-Pt nanoparticles with *S. aureus* and *E. coli*, respectively. **a** CLSM images of *E. coli* and *S. aureus* treated with Pd-Pt and EV-Pd-Pt (Pd-Pt concentration of 50 μg/mL) for 30 min, respectively. **b** CLSM tomography images of *E. coli* and *S. aureus* after 30 min incubation with Pd-Pt and EV-Pd-Pt nanoparticles, respectively. Red and green fluorescent signals represent Cy 5.5-labeled nanoparticles and SYTO 9-stained bacteria, respectively. Experiments were repeated three times independently with similar results.

The lipid bilayer structure of ginger-derived EVs is thought to be composed of phospholipids and glycerol lipids^31,40^. Among them, phosphatidic acid (PA) plays an important role in the uptake of ginger-derived EVs by *Lactobacillus rhamnosus* and *Porphyromonas gingivalis*, which is involved in vesicular endocytosis and could interact with proteins expressed on the bacterial surface^35,36^. These findings inspired us to investigate whether ginger-derived EV-functionalized Pd-Pt nanosheets (EV-Pd-Pt) could be taken up by bacteria. Gram-positive *S. aureus* and Gram-negative *Escherichia coli* (*E. coli*) were selected as model bacteria. To evaluate the internalization of EV-Pd-Pt, the localization of EV-Pd-Pt was monitored visually by confocal laser scanning microscope (CLSM) while Pd-Pt nanosheets were employed as counterparts. The interaction of EV-Pd-Pt and Pd-Pt with the SYTO 9-stained bacteria (green fluorescence) was tracked by the red fluorescence of Cy5.5 (Supplementary Fig. 8). As shown in Fig. 4a, after co-cultivation for 30 min, obvious red fluorescence from EV-Pd-Pt and merged yellow color of red and green fluorescence were observed inside both *S. aureus* and *E. coli*, indicating that EV-Pd-Pt could effectively intercalate into bacterial membranes and enter the cytoplasm. In contrast, the bacteria incubated with Pd-Pt displayed negligible red fluorescence, validating the hypothesis that ginger-derived EVs could greatly facilitate nanoparticle-bacteria interactions and promote the uptake of EV-Pd-Pt in an EV lipid-dependent manner. Furthermore, tomography imaging of *E. coli* and *S. aureus* were performed by CLSM to visualize the distribution of nanoparticles inside the bacteria (Fig. 4b). After 30 min of incubation, the red fluorescence of EV-Pd-Pt appeared not only around the bacterial membranes, but also inside the bacteria, confirming that EV-Pd-Pt entered the bacterial cytoplasm through the membrane.

In addition, the uptake of EV-Pd-Pt was studied by TEM imaging of the bacterial sections. As exhibited in Supplementary Fig. 9, a few EV-Pd-Pt nanoparticles were clearly found in the cytoplasm of *S. aureus*, confirming their internalization into bacterial cells. Furthermore, the uptake of EV-Pd-Pt and Pd-Pt by bacteria was quantified by flow cytometry and inductively coupled plasma-mass spectrometry (ICP-MS). As shown in Supplementary Fig. 10, significantly enhanced accumulation of EV-Pd-Pt in bacteria was found compared with Pd-Pt, validating the important role of EVs in preferential uptake.

The PA lipids in ginger-derived EVs are considered to serve as a signal for preferential uptake of EV-Pd-Pt, as depletion of PA lipids has been validated to lead to a significant reduction in uptake by *Lactobacillus rhamnosus*^35^. To further explore the mechanism of the enhanced uptake of EV-Pd-Pt by *E. coli* and *S. aureus*, total lipids from the ginger-derived EVs were extracted with chloroform and then separated by thin-layer chromatography (TLC)^35,36^. PA-depleted lipids were also obtained by the removal of PA band from the TLC according to standard PA migration. Thereafter, total lipids and PA-depleted lipids were reassembled into nano-sized vesicles (NVs), respectively. As displayed in Supplementary Fig. 11, NVs formed from PA-depleted lipids demonstrated evidently reduced uptake by *E. coli* and *S. aureus*. These results verify the significance of PA lipids in the enhanced uptake of EV-Pd-Pt. Taken together, the EV-Pd-Pt nanoparticles could be successfully taken up by bacteria in an EV lipid-dependent manner, which guarantees their accumulation and strong interaction with bacteria for enhanced EDT and antibacterial activity.

### Biocompatibility of EV-Pd-Pt nanoparticles

Since the biocompatibility of biomaterials is prerequisite for antibacterial applications, hemolysis and methylthiazolyl tetrazolium (MTT) assays were used to study the hemocompatibility and cytotoxicity of EV-Pd-Pt. The hemolysis ratio of EV-Pd-Pt solutions (12.5–200 μg/mL) was much lower than the permissible limit of 5%, indicating little hemolysis of red blood cells^6^ (Supplementary Fig. 12a). To investigate the cytotoxicity of EV-Pd-Pt, the viability of L929 cells was evaluated by MTT assay. As shown in Supplementary Fig. 12b, the cell viability remained above 90% when the concentration of EV-Pd-Pt was as high as 400 μg/mL, indicating negligible cytotoxicity to L929 cells. The excellent biocompatibility of EV-Pd-Pt makes them suitable for antibacterial applications.

### Antibacterial activity of EV-Pd-Pt nanoparticles in vitro

**Fig. 5:**
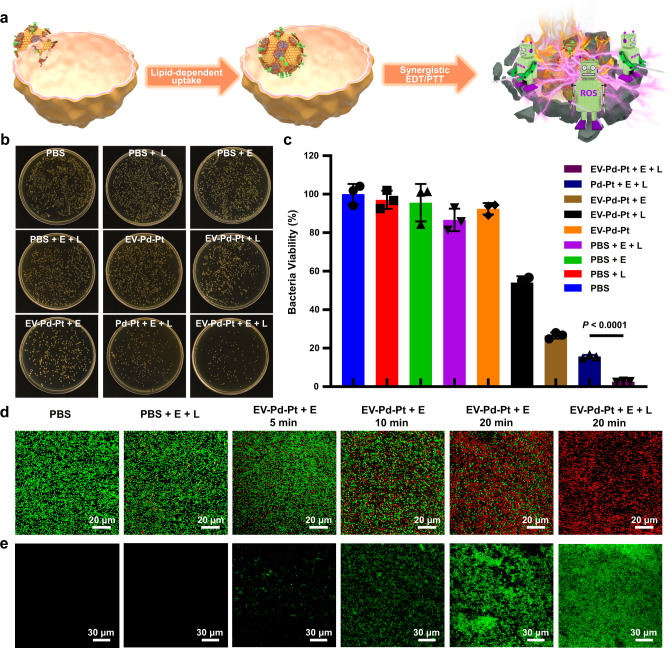
In vitro antibacterial activity of EV-Pd-Pt nanoparticles. **a** Schematic illustration of the uptake and antibacterial effect of EV-Pd-Pt. Killing effect of EV-Pd-Pt against *S. aureus* by the plate counting method. Photographs of *S. aureus* colonies (**b**) and corresponding viability (**c**) after different treatments. Data are presented as mean values ± SD (*n* = 3 independent samples). Statistical significance was calculated by two-tailed Student’s *t*-test. **d** Live/dead fluorescence staining images of *S. aureus* after different treatments. **e** Detection of ROS level in *S. aureus* after different treatments. **d**, **e** Experiments were repeated three times independently with similar results. Source data are provided as a Source Data file.

The desirable electro-driven catalytic activity, photothermal property, and biocompatibility of EV-Pd-Pt nanoparticles motivated us to explore their antibacterial effects. Benefiting from ginger-derived EVs, biomimetic EV-Pd-Pt nanoparticles exhibit unique characteristics that can be taken up by bacteria in a lipid-reliant manner. Therefore, it was speculated they could accumulate inside bacteria to show a strong bactericidal effect through combined EDT and PTT (Fig. 5a). The standard colony counting method was employed to quantitatively evaluate the antibacterial performance of EV-Pd-Pt (Fig. 5b, c). Compared with the PBS-cultured control group, the three groups of *S. aureus* treated with square-wave AC electric field, 980 nm NIR light irradiation, and their combination did not show obvious bacterial death, suggesting that neither square-wave AC field nor the NIR light had any killing effect on *S. aureus*. Similarly, *S. aureus* incubated with EV-Pd-Pt exhibited a high viability of over 90%. In contrast, the treatment of EV-Pd-Pt in combination with the electric field (EV-Pd-Pt + E, EDT group) significantly reduced bacterial viability, confirming that potent EDT triggered by EV-Pd-Pt could combat bacterial infection. In addition, moderate antibacterial activity of EV-Pd-Pt was observed after NIR light irradiation was performed (EV-Pd-Pt + L, PTT group), verifying the photothermal killing effect. As expected, the combination of electric field and NIR irradiation (EV-Pd-Pt + E + L, EDT/PTT group) induced the most severe bacterial death. Meanwhile, we determined the degree of synergy (*S*) using Bliss independence model^41^. A synergistic effect between EDT and PTT was observed in combined EDT/PTT antibacterial treatment (*S* > 0)^6^, which was due to the enhanced catalytic activity of EV-Pd-Pt under NIR irradiation. Notably, the bactericidal performance of EV-Pd-Pt produced by combined EDT/PTT was substantially higher than that of Pd-Pt, validating the hypothesis that the internalization of EV-Pd-Pt benefiting from ginger-derived EVs could promote the interaction between toxic ·OH and bacteria to achieve enhanced antibacterial activity. These results demonstrate the great potential of EV-Pd-Pt nanoparticles to eradicate bacterial infections through exploiting their intrinsic properties.

Additionally, the antibacterial effect of EV-Pd-Pt was intuitively demonstrated by live/dead staining assay. As exhibited in the CLSM images (Fig. 5d), live bacteria stained with SYTO-9 displayed green fluorescence while dead bacteria were labeled with propidium iodide (PI) in red. As expected, *S. aureus* in the PBS-treated groups exhibited predominantly green fluorescence. At the same time, evident bacterial death was observed in the EV-Pd-Pt treatment groups under the square-wave AC field. Furthermore, bactericidal effect increased with the increase of the current duration time, probably due to the continuous production of ROS. Upon NIR irradiation, nearly all bacteria displayed red fluorescence in the EV-Pd-Pt + E + L treatment group, indicating the excellent antibacterial effect of the synergistic EDT/PTT.

To reveal the antibacterial mechanism of EDT, the fluorescent probe 2′,7′-dichlorofluorescein diacetate (DCFH-DA) was employed to visualize ROS generation in *S. aureus* with different treatments. After the oxidation reaction with ROS, green fluorescence appeared due to the generation of 2′,7′-dichlorofluorescein. As shown in Fig. 5e, hardly any green fluorescence was observed in the presence or absence of an external electric field, indicating negligible level of ROS and little effect of electric field stimulation on ROS production. However, a weak green fluorescence signal could be found in *S. aureus* treated with EV-Pd-Pt and square-wave AC for 5 min. As the current duration time increased to 20 min, a sustained increase in ROS levels was observed, which could explain the corresponding enhancement in bactericidal performance of EV-Pd-Pt (Fig. 5d). After NIR light was applied, elevated levels of ROS were detected in the EV-Pd-Pt + E + L group, which is consistent with the NIR-enhanced catalytic activity of the Pd-Pt nanosheets (Fig. 3a)^42^. These results demonstrate the great potential of EV-Pd-Pt to combat bacterial infections through the synergistic effect of EDT and PTT.

### Antibiofilm effect of EV-Pd-Pt nanoparticles in vitro

**Fig. 6:**
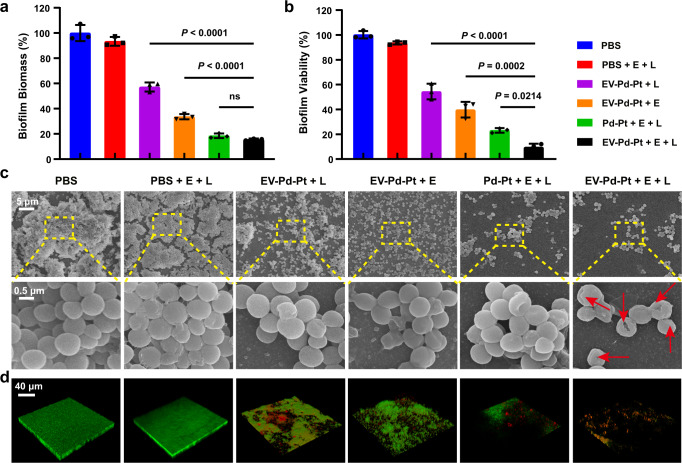
Antibiofilm activity of EV-Pd-Pt nanoparticles. Biomass (**a**) and corresponding viability (**b**) of *S. aureus* biofilms after different treatments. Data are presented as mean values ± SD (*n* = 3 independent samples). Statistical significance was calculated by one-way analysis of variance (ANOVA). Representative SEM images (**c**) and 3D CLSM of live/dead staining (**d**) of *S. aureus* biofilms after different treatments. The frequency of the square-wave AC electric field was 10 mHz, and the power density of NIR laser was 0.5 W/cm^2^. Source data are provided as a Source Data file.

It has been revealed that more than 80% of chronic infections are associated with biofilms^43^, which has become a severe threat in the clinic. More seriously, the treatment of biofilm-associated infections is challenging since the interior bacteria are protected by extracellular polymeric substances against the host immune attack and penetration of antibacterial agents. Encouraged by the excellent antibacterial activity of EV-Pd-Pt nanoparticles, we further investigated their capability against *S. aureus* biofilms by crystal violet staining assay and plate counting method. As shown in Fig. 6a, b, in the absence of EV-Pd-Pt nanoparticles, external stimulation with the square-wave AC electric filed and 980 nm NIR light did not noticeably attenuate biofilm biomass or reduce bacterial viability. In contrast, EV-Pd-Pt could eliminate *S. aureus* biofilms to some extent by the application of square-wave AC filed (EV-Pd-Pt + E, ~33.6%) or 980 nm NIR light irradiation (EV-Pd-Pt + L, ~57.2%). It is to be noted that the EV-Pd-Pt + E + L group showed a distinct decrease in both biofilm biomass (~15.6%) and bacterial viability (~9.6%) compared with monotherapy groups, indicating the excellent antibiofilm effect of EV-Pd-Pt to eradicate biofilms by the combination EDT/PTT. In addition, Pd-Pt-mediated EDT/PTT was also efficient in eliminating biofilms. Remarkably, a significant higher killing effect on *S. aureus* biofilm was found in the EV-Pd-Pt + E + L group compared with the Pd-Pt treatment group (Fig. 6b). These results suggest that EV-Pd-Pt nanoparticles are promising for biofilm elimination, accompanied by superior bactericidal performance resulting from efficient internalization capacity.

To further verify the destruction of *S. aureus* biofilms, the bacterial morphological changes of the biofilms were observed by scanning electron microscope (SEM). As shown in Fig. 6c, negligible antibiofilm effect was found in PBS-treated biofilms, which exhibited dense packing of *S. aureus* with intact spherical morphology. In contrast, the integrity of EV-Pd-Pt-incubated biofilms was disrupted after the application of square-wave AC filed or NIR light irradiation, verifying the antibiofilm effect of EV-Pd-Pt through photothermal ablation and ROS generation. As expected, the most severe disruption of the biofilm occurred after combined EDT/PTT was performed, where most bacteria wrinkled and distorted with damaged cell membranes (red arrows in Fig. 6c). These phenomena could be explained by the generation of ROS inside bacteria after EV-Pd-Pt nanoparticles were taken up. Additionally, under the stimulations of electric field and NIR light, the Pd-Pt-cultured biofilm displayed obvious dispersal, while most bacteria inside the biofilm maintained intact morphology, which is consistent with the antibiofilm and killing effect of Pd-Pt (Fig. 6a, b).

Furthermore, live/dead staining was employed to visualize the antibiofilm effect of EV-Pd-Pt. As exhibited in Fig. 6d, three-dimensional (3D) CLSM displays the corresponding fluorescent images of biofilms with different treatments. PBS-treated presented intact and dense biofilms with green fluorescence. Compared with biofilms treated with PTT or EDT alone with a large amount of green florescence, the biofilm treated with EV-Pd-Pt-mediated synergistic EDT/PTT showed only fewer red fluorescent spots, indicating more severe biofilm dispersal and bacterial death. Therefore, the potent biofilm dispersal ability and killing effect of EV-Pd-Pt under external electric field and NIR light stimulation were verified. The green fluorescence in the Pd-Pt + E + L group further indicates the inadequate bactericidal effect of Pd-Pt and the superiority of EV-mediated internalization. Taken together, EV-Pd-Pt nanoparticles could effectively eliminate biofilms and hold great potential in combating biofilm-associated infections through synergistic effect of EDT and PTT.

### In vivo imaging and biodistribution of EV-Pd-Pt nanoparticles

**Fig. 7:**
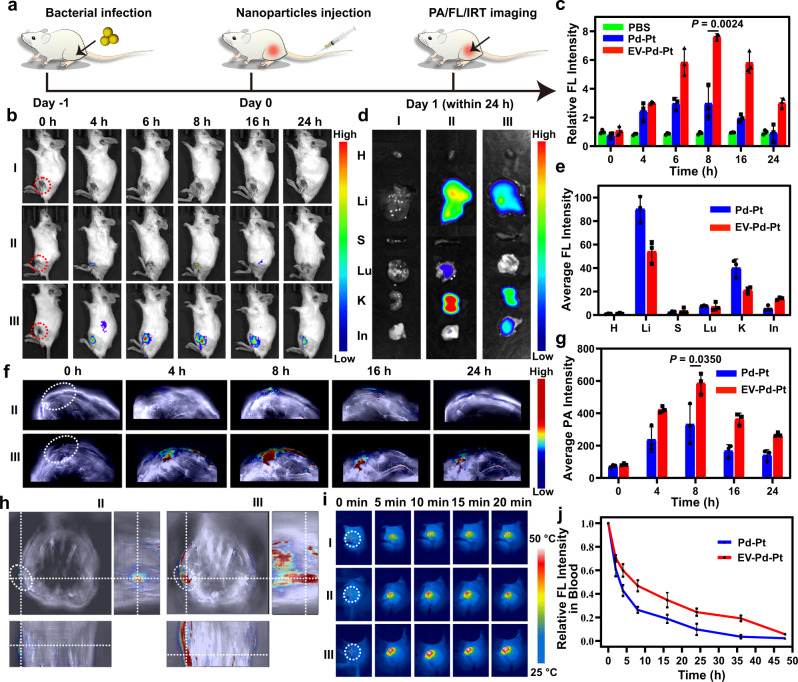
In Vivo imaging and biodistribution of EV-Pd-Pt nanoparticles. **a** Schematic illustration of the *S. aureus* infection model establishment for FL, PA, and IRT imaging. **b** In vivo time-dependent FL images of *S. aureus*-infected mice after the intravenous injection of PBS (I, control), Pd-Pt (II), and EV-Pd-Pt (III). **c** Quantitative analysis of *S. aureus*-infected thighs after different treatments. FL images (**d**) and average FL intensity (**e**) of major organs and the infected tissues after different treatments at 8 h post-injection. H, Li, S, Lu, K, and In represent heart, liver, spleen, lung, kidney, and infected tissue, respectively. PA images (**f**) and intensity (**g**) of *S. aureus*-infected thighs of mice after the intravenous injection of Pd-Pt (II) and EV-Pd-Pt (III), respectively. **h** 3D PA images of *S. aureus*-infected thighs from mice 8 h after injection of Pd-Pt (II) and EV-Pd-Pt (III), respectively. **i** IRT images of *S. aureus*-infected mice exposed to a 980 nm NIR laser (0.5 W/cm^2^) for 20 min after intravenous injection of PBS (I), Pd-Pt (II), and EV-Pd-Pt (III), respectively. **j** Time-dependent FL intensity of blood from *S. aureus*-infected mice after injection of Pd-Pt and EV-Pd-Pt, respectively. **c**, **e**, **g**, **j** Data are presented as mean values ± SD (*n* = 3 independent samples). **c**, **g** Statistical significance was calculated by two-tailed Student’s *t*-test. Source data are provided as a Source Data file.

EV-Pd-Pt nanoparticles are expected to be utilized in the treatment of bacterial infections in vivo due to their satisfactory antibacterial activity in vitro. Considering the unique internalization and long blood circulation time introduced by EVs, the in vivo accumulation of biomimetic EV-Pd-Pt nanoparticles at infection sites was firstly studied with *S. aureus*-infected mice. After intravenous injection, the biodistribution of EV-Pd-Pt nanoparticles in mice with subcutaneous abscesses at different time points was visually monitored by noninvasive fluorescence (FL), photoacoustic (PA), and infrared thermal (IRT) imaging (Fig. 7a). PBS and Pd-Pt nanoparticles were also administrated from the tail vein for comparison. As shown in Fig. 7b, no autofluorescence was observed in the PBS group (I) while weak FL signals appeared at the infection site after Pd-Pt nanoparticles were injected (II). In contrast, much stronger and persistent FL signals were detected at the infection site in the EV-Pd-Pt-treated group (III) during the 24 h monitoring period, indicating the efficient accumulation and long circulation of EV-Pd-Pt nanoparticles. In addition, the accumulation behavior of nanoparticles in the infected region was found to be time-dependent. Quantitative analysis of FL intensity confirmed that EV-Pd-Pt nanoparticles could accumulate at the infected sites better than Pd-Pt, and the maximum appeared at 8 h post-injection (Fig. 7c). The distribution of nanoparticles in the main organs and infected tissues at 8 h post-injection further demonstrated that EV-Pd-Pt could be effectively delivered to the infection site with a long retention time (Fig. 7d, e). Interestingly, strong FL signals were observed in the kidneys of both Pd-Pt and EV-Pd-Pt-treated groups, implying that the nanoparticles could be excreted via renal clearance. The corresponding quantitative analysis of Pd-Pt and EV-Pd-Pt nanoparticles in different organs with ICP-MS is consistent with the results of FL imaging (Supplementary Fig. 13).

Taking advantage of the photothermal properties of Pd-Pt, PA imaging of *S. aureus*-infected mice was performed and similar results were obtained (Fig. 7f, g). EV-Pd-Pt nanoparticles were distributed in the infected tissues with the highest PA intensity at 8 h after intravenous injection, which was 1.76-fold higher than that of the Pd-Pt-injected group. The 3D reconstruction of PA signal around the infection sites further demonstrated the bacteria targeting and specificity of EV-Pd-Pt nanoparticles (Fig. 7h). Additionally, the excellent photothermal capacity of Pd-Pt endowed EV-Pd-Pt with the IRT imaging ability. As shown in Fig. 7i and Supplementary Fig. 14, at 8 h post-injection, the IRT signal and corresponding temperature at the site of infection gradually increased with irradiation time. Meanwhile, after 20 min of 980 nm NIR laser irradiation, the temperature elevation of the infected tissue of the mice administrated with EV-Pd-Pt was much higher (~53 °C) than that of Pd-Pt or PBS-injected groups, demonstrating the feasibility of EV-Pd-Pt for PTT in vivo. The in vivo FL, PA and IRT visualization of the infection sites were mainly attributed to the excellent bacteria targeting ability and long circulation of the biomimetic nanoparticles. Taken together, precise and accurate information could be provided by these imaging modalities, which not only validates the effective accumulation of EV-Pd-Pt nanoparticles at infection sites, but also provides valuable guidance for the treatment of bacterial infections in vivo.

To study the clearance behavior of EV-Pd-Pt nanoparticles in vivo, Cy5.5-labeled nanoparticles were intravenously injected, and FL quantification was performed on blood samples obtained at different time points. As shown in Fig. 7j, EV-Pd-Pt nanoparticles exhibited much higher FL intensities in blood than Pd-Pt within 50 h, suggesting their prolonged blood circulation benefiting from ginger-derived EVs. Additionally, the FL signals in urine and feces were investigated to track the metabolism and excretion pathways of EV-Pd-Pt. As demonstrated in Supplementary Fig. 15, both hepatic metabolism and renal clearance occurred after intravenous administration of nanoparticles. Meanwhile, the clearance rate of Pd-Pt was much faster than that of EV-Pd-Pt, which is consistent with the long circulation and retention of EV-functionalized nanoparticles. More importantly, the excretion results indicate that biomimetic EV-Pd-Pt nanoparticles were metabolizable and could avoid potential side effects, further ensuring their biosafety for in vivo applications.

### Antibacterial activity of EV-Pd-Pt nanoparticles in vivo

**Fig. 8:**
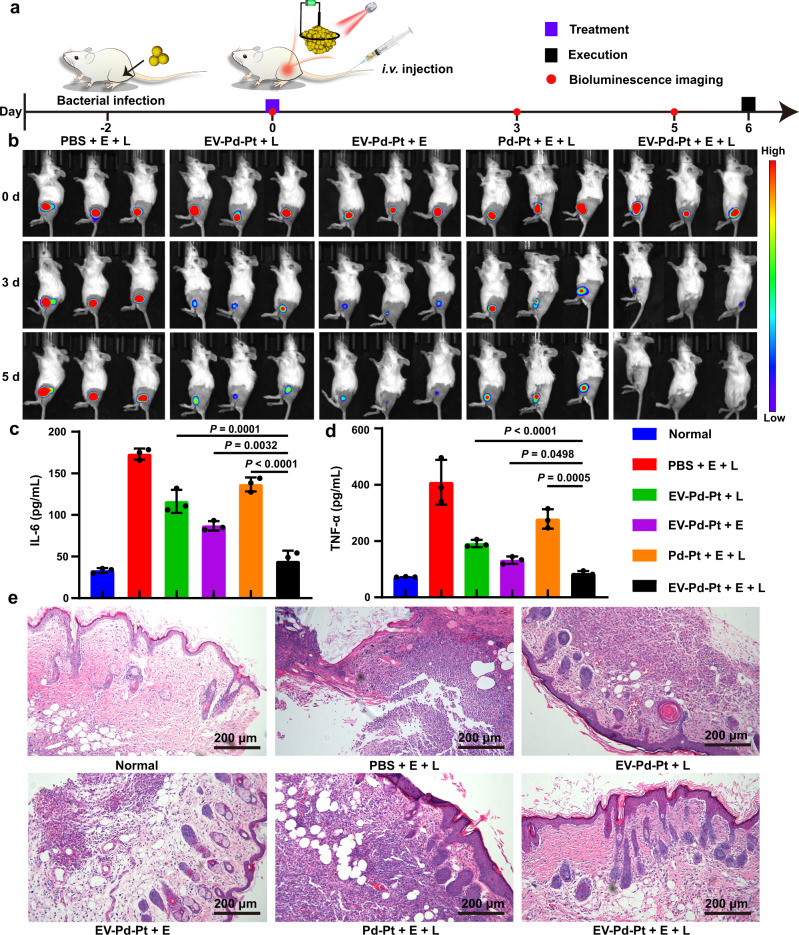
In vivo antibacterial effect of EV-Pd-Pt on the luciferase-expressing *S. aureus*-infected mice model. **a** Schedule of the infection, systemic treatment, and monitoring of subcutaneous abscess in mice with bioluminescence imaging after intravenous (i.v.) injection. **b** Bioluminescence images of *S. aureus*-infected mice with different treatments on days 0, 3, and 5. Quantitative analysis of inflammatory cytokines including IL-6 (**c**) and TNF-*α* (**d**) after 5 days. Data are presented as mean values ± SD (*n* = 3 independent samples). Statistical significance was calculated by one-way ANOVA. **e** H&E staining images of the infected tissues from abscess sites with various treatments after 5 days. Experiments were repeated three times independently with similar results. Source data are provided as a Source Data file.

Encouraged by the excellent biocompatibility, antibacterial activity in vitro, and high accumulation ability of EV-Pd-Pt nanoparticles, we investigated the antibacterial performance of EV-Pd-Pt nanoparticles in vivo. To visualize the therapeutic effect, a mouse infection model was established using luciferase-expressing *S. aureus*. Two days later, *S. aureus*-infected mice were intravenously injected with PBS or nanoparticles via the tail vein, and bioluminescence imaging was performed to monitor the in vivo antibacterial efficiency of different treatments (Fig. 8a). Representative bioluminescence images of *S. aureus*-infected mice 5 days post-injection are exhibited in Fig. 8b. Apparently, the PBS treatment with the application of square-wave AC field and NIR light irradiation failed to combat *S. aureus* infection. In contrast, less intense bioluminescence signals were observed in the EV-Pd-Pt-mediated PTT, EDT, and Pd-Pt-mediated EDT/PTT groups after 5 days, indicating their antibacterial effects to some extent. Notably, a negligible bioluminescence signal appeared in the EV-Pd-Pt-mediated EDT/PTT group, demonstrating eradication of the subcutaneous abscess. In addition, bacteria at infection sites after different treatments were cultured and quantified by the plate counting method (Supplementary Fig. 16), indicating that the antibacterial performances were consistent with the bioluminescence imaging. It was found that combined EDT/PTT mediated by EV-Pd-Pt exhibited the least number of colonies while a synergistic antibacterial effect was achieved between EDT and PTT in vivo (*S* > 0)^6^. Interestingly, the synergistic EDT/PTT mediated by EV-Pd-Pt demonstrated much better bactericidal effect than Pd-Pt-mediated EDT/PTT, verifying the excellent in vivo antibacterial activity of EV-Pd-Pt benefiting from prolonged blood circulation, efficient accumulation at infection sites, and uptake by bacteria.

Furthermore, the expression of inflammatory cytokines including tumor necrosis factor-α (TNF-*α*) and interleukin-6 (IL-6) in the serum of infected mice were analyzed by enzyme-linked immunosorbent assay (ELISA). As displayed in Fig. 8c, d, the cytokine levels of TNF-*α* and IL-6 in EV-Pd-Pt-mediated synergistic EDT/PTT group were significantly lower than those in other treatment groups after 5 days, which almost returned to the levels of normal mice. These results suggest that the synergistic EDT/PTT could efficiently combat bacterial infection and relieve inflammation in vivo. As shown in Fig. 8e, hematoxylin and eosin (H&E) staining was also performed to visualize the inflammatory cells and evaluate the antibacterial effect of different treatments. Numerous inflammatory cells could be observed in the PBS + E + L group, suggesting the existence of severe bacterial infection. In contrast, fewer inflammatory cells were found in the Pd-Pt-mediated PTT, EDT, and Pd-Pt-mediated EDT/PTT groups. As expected, EV-Pd-Pt-mediated EDT/PTT group displayed negligible inflammatory cells, further confirming the superiority of synergistic bacterial killing effect.

Moreover, the corresponding histological analysis of major organs, including heart, liver, spleen, lung, and kidney collected from sacrificed mice after 5 days of treatment did not show noticeable inflammatory or abnormal lesions (Supplementary Fig. 17). In addition, the negligible change in body weight during the treatment period indicates the compromised side effect of the nanoparticles in vivo (Supplementary Fig. 18). Also, blood biochemical indexes of the EV-Pd-Pt + E + L group including the white blood cells, red blood cells, platelet count, and hemoglobin did not show noticeable difference from the normal group (Supplementary Fig. 19), validating the excellent biosafety and biocompatibility of EV-Pd-Pt-mediated synergistic EDT/PTT. Taken together, biomimetic EV-Pd-Pt nanoparticles are promising for safe and efficient antibacterial therapy in vivo.

## Discussion

In conclusion, a facile strategy was proposed to fabricate biomimetic nanoplatform (EV-Pd-Pt) for synergistic anti-infective therapy. Pd-Pt nanosheets with intrinsic electrodynamic and photothermal properties were firstly synthesized. The enhanced electro-driven catalytic activity of Pd-Pt over Pt nano-electrosensitizers was verified by both experimental data and theoretical calculations. Then ginger-derived EVs were conjugated with Pd-Pt nanosheets to successfully fabricate EV-Pd-Pt nanoparticles. Ginger-derived EVs endowed EV-Pd-Pt nanoparticles with excellent biocompatibility and long blood circulation, which ensured the efficient accumulation of nanoparticles at infection sites. More importantly, EV-Pd-Pt nanoparticles were proved to be taken up by bacteria in an EV-dependent manner, which achieved strong interactions with bacteria to enhance the bactericidal effect. In particular, the short lifetime and diffusion distance limitations of ROS could be overcome by sustainable in situ generation of ROS inside bacteria. Notably, a synergistic bactericidal effect between EDT and PTT was achieved, which is potent to combat bacterial and biofilm infections regardless of the microenvironment. In addition, versatile imaging including PA, FL, and IRT imaging was performed to visualize the accumulation and excretion of nanoparticles in vivo. Finally, a luciferase-expressing *S. aureus* infection mouse model was established to verify the in vivo antibacterial effect of EV-Pd-Pt nanoparticles. The favorable biocompatibility of the metabolizable EV-Pd-Pt and their excellent bactericidal performance benefiting from synergistic EDT/PTT suggest their feasibility for the treatment of bacterial infections in vivo. The current work provides a promising avenue for facile construction of biomimetic nanoparticles for synergistic anti-infective therapy in complex in vivo infection environments.

## Methods

### Materials

Palladium (II) acetylacetonate (Pd(acac)_2_), potassium tetrachloroplatinate (K_2_PtCl_4_), tungsten hexacarbonyl (W(CO)_6_), hexadecyltrimethylammonium bromide (CTAB), polyvinylpyrrolidone (PVP, MW: 40000), 2,7-dichlorofluorescein diacetate (DCFH-DA), and 3-(4,5-Dimethylthiazol-2yl)-2,5-diphenyl tetrazolium bromide (MTT) were purchased from Sigma-Aldrich Chemical Co. (St Louis, MO, USA). Ascorbic acid (AA) was obtained from Sinopharm Group Co. Ltd (China). Lipoic acid (LA), 2-(N-morpholino) ethanesulfonic acid (MES), and citric acid (CA), N-hydroxysuccinimide (NHS), 2,2,6,6-tetramethylpiperidine (DMPO), 1-ethyl-3-[3-(dimethylamino)propyl] carbodiimide hydrochloride (EDC), and methylene blue (MB) were provided by Energy Chemical (Shanghai, China). Thiol-modified polyethylene glycol (HS-PEG) was obtained from PonsureBio (Shanghai, China). *N, N*-dimethylformamide (DMF), dimethyl sulfoxide (DMSO, >90%), acetone (>99.5%), ethanol (>99.7%), and sodium hydroxide (NaOH) were provided by Beijing Chemical Works (China). All the reagents were analytical reagent grade and used without further purification. The lysogeny broth (LB) solution was autoclaved for 20 min at 120 °C before use. The strains of *E. coli* (ATCC 25922) and *S. aureus* (CMCC (B) 26003) were obtained from Promega (Madison, USA). The LIVE/DEAD BacLight Bacterial Viability Kit (L7012) was purchased from Invitrogen (LifeTechnologies, USA).

### Synthesis of Pd nanosheets

Typically, 16 mg of Pd(acac)_2_, 30 mg of PVP, 60 mg of CTAB, and 170 mg of CA were mixed in a glass flask containing 10 mL of DMF. The mixture was stirred at room temperature for 1 h to obtain a homogeneous orange-red solution. 100 mg of W(CO)_6_ was subsequently added. The mixture was then heated to 80 °C in N_2_ atmosphere and kept under magnetic stirring for 1 h. After cooling down to room temperature, the resultant Pd nanosheets were precipitated using acetone, collected by centrifugation at 5000 g, and re-dispersed in deionized water for subsequent experiments.

### Synthesis and functionalization of Pd-Pt nanosheets

The Pd-Pt nanosheets were synthesized by a seed-mediated method^37,38^. Briefly, 101.75 mg of PVP and 600 mg of AA were mixed with 5 mL of the as-synthesized Pd nanosheet suspension. Then, the mixture was stirred at room temperature for 40 min, and 0.5 mL of K_2_PtCl_4_ aqueous solution (100 mM) was added. The mixture was vigorously stirred for another 30 min at room temperature and then immediately transferred to a 90 °C oil bath for 4 h. After cooling down to room temperature, the Pd-Pt nanosheets were collected and washed with distilled water several times. To functionalize the Pd-Pt nanosheets, dihydrolipoic acid (DHLA) was firstly synthesized. 30 g of LA was added to 100 mL of anhydrous ethanol and the pH of the solution was adjusted to ~9 with NaOH solution. Then sodium borohydride was added to trigger the ring opening of the dithiolane. After stirring at room temperature for 1 h, DHLA was obtained by filtration and evaporation. Then, 20 mg of SH-PEG and DHLA (molar ratio = 5:1) was added in the above Pd-Pt nanosheet solution for carboxyl functionalization and improved stabilization. Finally, the solution was kept stirring for 12 h, and the carboxyl-functionalized Pd-Pt nanosheets were collected by centrifugation at 18,000 *g* for 20 min and stored at 4 °C for subsequent use.

### Isolation and purification of EVs

The EVs derived from ginger were extracted from fresh ginger roots^32,44^. Firstly, the fresh ginger roots were purchased from a local store and washed thoroughly with deionized water. The ginger was minced, soaked in ice-cold phosphate-buffered saline (PBS, 10 mM, pH 7.4), and then ground in a blender to obtain ginger juice. Blended ginger juice was sequentially centrifuged at 1000 *g* for 10 min, 3000 *g* for 20 min, and 10,000 *g* for 40 min at 4 °C to remove large particles and fiber fragments. The supernatant was collected for further ultracentrifugation (Beckman Optima XE-100, Beckman, USA) at 150,000 g for 2 h. Then, the pellet was resuspended in PBS and transferred to a sucrose gradient (8%, 15%, 30%, 45%, and 60%), followed by centrifugation at 150,000 *g* for 2 h to isolate and purify EVs. The bands between the 8%/30% layer and 30%/45% layer were harvested separately and then washed with PBS, followed by centrifugation at 150,000 *g* for 1 h at 4 °C. The obtained EVs were then resuspended in PBS and stored at −80 °C for further use. The mass of EVs was determined by weighing after lyophilization.

### Preparation of EV-Pd-Pt nanoparticles

Briefly, 1.0 mg of functionalized Pd-Pt nanosheets were dispersed in 2 mL of MES buffer (pH 6.0), and then 6.0 mg of EDC and 6.0 mg of NHS were added to activate the -COOH groups. After being stirred at room temperature for 2 h, the prepared EVs (5.0 mg) were added, and the solution was shaken overnight. Finally, the EV-Pd-Pt were harvested by ultracentrifugation at 8000 *g* for 20 min and re-dispersed in PBS buffer. The content of Pd-Pt nanosheets in EV-Pd-Pt was determined by using a Tarsus TG 209 F3 thermogravimetric analyzer (Netzsch, Germany). The thermogravimetric measurements were carried out in air from room temperature to 850 °C at a heating rate of 10 °C/min.

### Electro-driven catalytic activity of Pd-Pt nanosheets

The electro-catalytic activity of Pd-Pt nanosheets was evaluated by analyzing the degradation rates of MB^18–21^. Typically, 2 mL of an aqueous solution containing Pd-Pt nanosheets (50 μg/mL) and MB (3 × 10^−5^ M) was placed in a cell of a 24-well plate, which was equipped with symmetrically arranged platinum electrodes and salt bridges (Φ = 3 mm, Saturated KCl). The two electrodes were connected with square-wave AC with different frequencies (i.e., 10, 50, 150, and 300 mHz). At fixed time intervals, 50 μL of the mixed solution was extracted to measure the absorption of MB.

In addition, the ·OH generation was verified by ESR spectra. Briefly, a 1 mL solution (50 μg/mL) of Pd-Pt nanosheets or a mixture of Pd nanosheets and Pt nanoparticles with the equivalent Pd and Pt contents was added into the solution containing 10 μL of DMPO, respectively. Then, the mixture was exposed to the square-wave AC field with 10 mHz for 30 min, and the characteristic signals were recorded on an ELEXSYS-II E500 ESR spectrometer (Bruker, Germany).

### Calculation method

In this work, structural optimization was performed by using first-principles calculations within the framework of DFT, as implemented in the plane wave set Vienna ab initio Simulation Package (VASP) code. The exchange-correlation energy was modeled by using Perdew-Burke-Ernzerhof (PBE) functional within the generalized gradient approximation (GGA). A cutoff energy of 400 eV was adopted. A Gaussian smearing of 0.02 eV to the orbital occupation was applied during the geometry optimization and for the total energy computations. The k-point sampling of the Brillouin zone was obtained using a 3 × 3 × 1 grid for the repetitive unit. A large vacuum slab of 15 Å was inserted in the z-direction for surface isolation to prevent interaction between two neighboring surfaces. A four-layer supercell was modeled for Pt(111), Pd(100), and Pd(100)-Pt (ten atoms of Pt(111) on Pd(100)) respectively. The upper two atomic layers of the slab were allowed to relax freely, while the bottom two atomic layers were fixed to simulate the bulk phase. Activation barriers for elementary steps of hydrolysis were determined by the climbing image nudged elastic band method (CI-NEB). The adsorption energy of H_2_O adsorbed on the surface model is defined as:

1 $${E}_{{{{{{\rm{ad}}}}}}}={E}_{{{{{{{\rm{H}}}}}}}_{2}{{{{{\rm{O}}}}}}@{{{{{\rm{slab}}}}}}}-{E}_{{{{{{\rm{slab}}}}}}}-{E}_{{{{{{{\rm{H}}}}}}}_{2}{{{{{\rm{O}}}}}}}$$

Among them, ${E}_{{{{{{{\rm{H}}}}}}}_{2}{{{{{\rm{O}}}}}}@{{{{{\rm{slab}}}}}}}$ is the total energy of H_2_O@slab (slab = Pt(111), Pd(100), and Pd(100)-Pt). E_slab_ is the energy of the surface model and is the energy of H_2_O.

### Photothermal property of Pd-Pt nanosheets

To investigate the photothermal performances of Pd-Pt, PBS buffers containing different concentrations (0, 25, 50, 100, and 200 μg/mL) of Pd-Pt were continuously exposed to 980 nm laser irradiation at a power density of 0.5 W/cm^2^ for 40 min. The temperatures were recorded by an infrared thermal imaging camera (FLIR Systems Inc., Ohio, USA) at predetermined time points.

### Uptake of Nanoparticles by bacteria

*E. coli* (ATCC 25922) and *S. aureus* (CMCC (B) 26003) were employed for CLSM observation^45^. Pd-Pt-Cy5.5 was firstly prepared by conjugating fluorescent Cy5.5-NHS onto the surface of Pd-Pt. Briefly, 20 mg of SH-PEG-NH_2_ and DHLA (molar ratio = 5:1) were mixed in the above Pd-Pt solution and kept stirring for 12 h. The NPs were then dispersed in PBS (0.1 M, pH 8.5) after centrifugation, followed by the addition of 1.0 mg/mL Cy5.5-NHS, and the reaction mixture was stirred at 4 °C in the dark for 24 h. Finally, the resulting Pd-Pt-Cy5.5 was purified by centrifugation. Cy5.5-labeled EV-Pd-Pt was prepared by conjugating Pd-Pt-Cy5.5 onto the surface of EVs. The conjugation with Cy5.5 was confirmed by Xenogen IVIS Spectrum (Caliper Life Sciences, America) and fluorescence spectroscopy (Hitachi F-7000) with excitation at 640 nm, respectively. Thereafter, Cy5.5-labeled EV-Pd-Pt and Pd-Pt (Pd-Pt concentration of 100 μg/mL, 0.5 mL) were cultured with bacterial suspensions (2 × 10^8^ CFU/mL, 0.5 mL) for 30 min. The bacteria were stained with SYTO 9 (6 μmol/mL in PBS) and the samples were observed by CLSM (Leica, SP8). For TEM imaging of the bacterial sections, the bacteria (2 × 10^8^ CFU/mL, 0.5 mL) were incubated with EV-Pd-Pt (Pd-Pt concentration of 100 μg/mL, 0.5 mL) for 30 min, washed with PBS twice, and fixed overnight using 4% paraformaldehyde at room temperature. Then secondary fixation with 1% osmium tetraoxide and dehydration in a gradient ethanol series were performed. The bacteria were finally embedded in Epon resin after polymerization overnight. Embedded samples were sectioned (100 nm in thickness) and examined under a transmission electron microscope (HT-7800, Hitachi, Japan). In addition, the uptake of EV-Pd-Pt and Pd-Pt nanoparticles was quantified by flow cytometry (BD Accuri C6 plus, USA) and by FlowJo software. The Pd content in bacteria was determined by ICP-MS (Agilent 7700).

### Cytotoxicity assay

3-(4,5-dimethylthiozol-2-yl)-2,5-diphe-nyltetrazolium bromide (MTT) assay was employed to evaluate the cytotoxicity of the resultant EV-Pd-Pt. In this work, L929 cells were seeded into 96-well plates at a density of 1 × 10^4^ cells per well, and cultured for 24 h at 37 °C. Then, the medium was replaced with a fresh culture medium (100 μL) containing EV-Pd-Pt with different concentrations. After being co-cultured for 24 h, 10 μL of MTT solution (5 mg/mL) were added to each well and then incubated for another 4 h. Finally, 100 μL of DMSO was transferred to each well to produce formazan crystals, and the absorbance values at 490 nm were recorded using a Bio-Rad Model 680 Microplate Reader (UK).

### Hemolysis analysis

The blood from mice was suspended in PBS (10 mM, pH 7.4) and red blood cells (RBCs) were separated by centrifugation. The RBCs were then washed with PBS until the supernatant was clear. Thereafter, RBC stock dispersion (100 μL) were added into 1 mL of PBS (10 mM, pH 7.4) containing EV-Pd-Pt with different concentrations (200, 100, 50, 25, and 12.5 μg/mL), and the mixtures were incubated at 37 °C for 3 h. Pure water and PBS (10 mM, pH 7.4) were used as the positive control and negative control, respectively. After centrifugation at 425 *g* for 15 min, the absorbance of the supernatant at 540 nm was measured. Finally, the hemolysis ratio was calculated by the following equation.

2 $${{{{{\rm{Hemolysis}}}}}}\,{{{{{\rm{ratio}}}}}} \, (\%)=\frac{{O}{{D}}_{{{{{{\rm{test}}}}}}}-{O}{{D}}_{{{{{{\rm{neg}}}}}}}}{{O}{{D}}_{{{{{{\rm{pos}}}}}}}-{O}{{D}}_{{{{{{\rm{neg}}}}}}}}\times 100\%$$

Where *OD*_test_ represents the absorbance of RBCs exposed to EV-Pd-Pt, *OD*_neg_ represents the absorbance of RBCs exposed to PBS (10 mM, pH 7.4), and *OD*_pos_ is the OD value of RBCs exposed to water.

### In vitro antibacterial assay

*S. aureus* (Gram-positive, CMCC (B) 26003) were employed as the representative bacteria to investigate the antibacterial effect of EV-Pd-Pt through the plate counting method^46,47^. Firstly, the bacteria were cultured aerobically in an LB medium at 37 °C for 12 h and harvested at the logarithmic growth phase. Then, 0.8 mL of bacterial suspension with a concentration of 10^6^ CFU/mL was treated with 0.2 mL of EV-Pd-Pt or Pd-Pt solution at a final Pd-Pt concentration of 50 μg/mL in the presence or absence of AC electric field (10 mHz, 20 min) and 980 nm NIR light irradiation (0.5 W/cm^2^, 20 min), respectively. The bacterial suspensions in different groups were then studied: PBS, PBS + L, PBS + E, PBS + E + L, EV-Pd-Pt, Pd-Pt + E + L, EV-Pd-Pt + L, EV-Pd-Pt + E, EV-Pd-Pt + E + L. Thereafter, the bacterial suspensions were diluted 50 times with PBS. Then 50 μL of diluted bacterial solution were spread on the LB agar plate and further incubated at 37 °C for 16 h to count the number of colonies.

### Live/dead bacterial staining analysis

The bacterial suspensions (10^6^ CFU/mL) was incubated with PBS (10 mM, pH 7.4) or EV-Pd-Pt (final concentration: 50 μg/mL) at 37 °C for 30 min, with or without square-wave AC treatment (10 mHz, different durations of 5, 10, and 20 min) and NIR irradiation (0.5 W/cm^2^, 20 min). Then, the bacterial suspension (10^9^ CFU/mL) was mixed with SYTO 9 and PI for 10 min. Finally, the stained bacteria were imaged using CLSM (Leica, SP8).

### ROS detection in bacteria

Briefly, *S. aureus* suspension at a concentration of 10^6^ CFU/mL was co-cultured with EV-Pd-Pt at 37 °C for 10 min. Subsequently, the bacteria were stained with DCFH-DA (10 μM), and treated with AC electric field (10 mHz) with or without NIR irradiation (0.5 W/cm^2^). After being washed three times by PBS (10 mM, pH 7.4), the bacteria (10^9^ CFU/mL) were imaged using CLSM (Leica, SP8).

### In vitro antibiofilm assay

Firstly, *S. aureus* was cultured in LB medium at 37 °C, and the biofilm was harvested in a confocal glass dish after 3 days of culture. Then, biofilms were randomly divided into seven groups for different treatments: (1) PBS, (2) PBS + E + L, (3) Pd-Pt + E + L, (4) EV-Pd-Pt, (5) EV-Pd-Pt + E, (6) EV-Pd-Pt + L, and (7) EV-Pd-Pt + E + L. The biofilms were then treated with EV-Pd-Pt or Pd-Pt solution at a Pd-Pt concentration of 100 μg/mL with or without AC electric field (10 mHz, 20 min) and 980 nm NIR light irradiation (0.5 W/cm^2^, 20 min), respectively. Crystal violet method was employed to quantify the biomass of these biofilms. Briefly, the biofilms were stained with 0.1% crystal violet for 10 min and washed twice with PBS (10 mM, pH 7.4). Finally, 500 μL of ethanol was added to the plate to extract crystal violet, and the absorbance at 595 nm was recorded by Bio-Rad Model 680 Microplate Reader (UK). In addition, the antibiofilm effect was quantified by standard plate counting assay. The biofilms were detached into sterile PBS (10 mM, pH 7.4) by sonication and vortexing. Thereafter, the diluted suspension was spread onto agar plates and the colony-forming units were counted after incubation at 37 °C for 16 h.

To realize live/dead observation, the biofilms were stained by the mixed SYTO 9/PI dyes and imaged using CLSM. For SEM imaging, the dishes containing *S. aureus* biofilm were fixed with 2.5% glutaraldehyde for 24 h at 4 °C, and dehydrated in gradient ethanol (50%, 75%, 90%, and 100%). After drying, the samples were sprayed with gold and observed by SEM (Zeiss Supra 55).

### *S. aureus* subcutaneous infection mouse model

All animal experiments were performed in compliance with the protocols approved by the Ethical Committee of the Chinese Academy of Medical Sciences and Peking Union Medical College. Female Balb/c mice (4–6 weeks old) were acquired from Beijing Vital River Laboratory Animal Technology Co., Ltd. (Beijing, China). The mice were anesthetized with isoflurane. The infection mouse model was established by injecting *S. aureus* suspension (3 × 10^8^  CFU/mL, 100 μL) into the right thigh. After 24 h, a subcutaneous abscess was created at the site of infection.

### In vivo FL imaging

The *S. aureus*-infected mice were intravenously injected by 200 μL of PBS (10 mM, pH 7.4), EV-Pd-Pt-Cy5.5 and Pd-Pt-Cy5.5 (Pd-Pt concentration of 2 mg/mL), respectively. The real-time FL imaging at predetermined time points (0, 4, 6, 8, 16, and 24 h) were carried out using a Xenogen IVIS Spectrum (Caliper Life Sciences, America). To evaluate the in vivo biodistribution of nanoparticles, the mice were sacrificed 8 h after injection and the main organs (heart, liver, spleen, lung, and kidney) and infected thigh tissues were collected for FL imaging.

### In vivo PA imaging

For in vivo PA imaging, EV-Pd-Pt or Pd-Pt nanoparticles (2 mg/mL, 200 μL) were intravenously injected into *S. aureus*-infected mice respectively. PA images were acquired 0, 4, 8, 16, and 24 h after the injection. A series of cross-section images were obtained using the multispectral optoacoustic tomography system (MSOT invision 256-TF, iThermedical, Germany).

### In Vivo IRT imaging

To investigate in vivo IRT imaging, 200 μL of EV-Pd-Pt or Pd-Pt solution (Pd-Pt concentration of 2 mg/mL) were injected into the mice intravenously, and PBS-treated group was regarded as a control. After 8 h, the infected regions were irradiated by a 980 nm laser (0.5 W/cm^2^) for 20 min. The temperature variations and images were recorded by an infrared thermal camera (FLIR Systems Inc., Ohio, USA) at predetermined time points during laser irradiation.

### In vivo blood circulation and excretion analysis of EV-Pb-Pt

The infected mice were injected intravenously with PBS (10 mM, pH 7.4), EV-Pd-Pt-Cy5.5, and Pd-Pt -Cy5.5 (at a concentration of 2 mg/mL), respectively. At different time points, the samples of blood, urine, and feces were collected and analyzed using a Xenogen IVIS Spectrum (Caliper Life Sciences, America).

### Antibacterial effect in vivo

The subcutaneous infected mice were prepared via subcutaneous injection with the luciferase-expressing *S. aureus* (100 μL, ~10^8^ CFU/mL). After 48 h, subcutaneous abscesses could be observed at the infected sites. Balb/c mice (4 weeks old) were randomly divided into five groups with seven mice in each group for different treatments: (1) PBS + E + L, (2) Pd-Pt + E + L, (3) EV-Pd-Pt + E, (4) EV-Pd-Pt + L, and (5) EV-Pd-Pt + E + L, respectively. 200 μL of PBS or nanoparticles at a Pd-Pt concentration of 2 mg/mL were injected intravenously via the tail vein, respectively. The treatment of square-wave AC field (10 mHz, 20 min) or 980 nm NIR (0.5 W/cm^2^, 20 min) irradiation were performed at 8 h post-injection. For bioluminescence imaging, 75 μL of luciferin (15 mg/mL, PerkinElmer, America) was injected to each mouse and the distribution of luciferase-expressing *S. aureus* was visualized at different post-injection time points through a Xenogen IVIS Spectrum (Caliper Life Sciences, America). The body weight of each mouse was recorded every day throughout the treatment. After 5 d, the blood samples from the EV-Pd-Pt + E + L group were collected for various blood-index measurements. Moreover, the inflammatory factors TNF-*α* and IL-6 in serum were analyzed by ELISA kits. Then all mice were sacrificed and the infected tissues were excised and homogenized in normal saline. 50 μL of diluted bacterial suspension was spread onto LB agar plate and cultured at 37 °C for 24 h. Then the number of colonies was counted. Major organs including heart, liver, spleen, lung, and kidney were harvested and fixed in 4% formaldehyde solution for H&E staining.

### Statistical analysis

Data were presented as means ± standard deviation and are from at least three independent experiments. The differences were calculated by using two-tailed Student’s *t*-test for two groups or one-way ANOVA for three or more groups followed by Dunnett’s multiple comparison post-hoc test. The statistical analysis were carried out with the sofeware of Excel 2019 or GraphPad Prism. The level of significance in all statistical analyses was set at *P* < 0.05.

### Reporting summary

Further information on research design is available in the Nature Portfolio Reporting Summary linked to this article.

## Supplementary information

Supplementary Information

Reporting Summary

## Data Availability

Data supporting the findings of this study are available within the article and the Supplementary Information. Source data are available for Figs. 2e, h–k, 3a–c, e, 5c, 6a, b, 7c, e, g, j, 8c, d, and Supplementary Figs. 3, 5, 6, 7b, 8, 10c, 12–15, 18 and 19 in the associated source data file. Other data are available from the corresponding authors upon reasonable request. Source data are provided with this paper.

## Source data

Source Data
