# Supplementary material for: Biomimetic electrodynamic nanoparticles comprising ginger-derived extracellular vesicles for synergistic anti-infective therapy
Source: Nat Commun. 2022 Nov 22;13:7164. doi: 10.1038/s41467-022-34883-5 (PMC9684156; doi:10.1038/s41467-022-34883-5)
Supplement: Supplementary file 1 — Supplementary Information [file 41467_2022_34883_MOESM1_ESM.pdf]

Supplementary Information

Biomimetic electrodynamic nanoparticles comprising ginger-derived extracellular vesicles for synergistic anti-infective therapy

Zhuangzhuang Qiao,[1,2,3,5] Kai Zhang,[1,2,3,5] Jin Liu,[4] Daojian Cheng[4], Bingran Yu,[1,2,3] Nana Zhao,[1,2,3]* & Fu-Jian Xu[1,2,3]*

[1]State Key Laboratory of Chemical Resource Engineering, Beijing University of Chemical Technology, Beijing 100029, China
[2]Key Laboratory of Biomedical Materials of Natural Macromolecules (Beijing University of Chemical Technology), Ministry of Education, Beijing 100029, China
[3]Beijing Laboratory of Biomedical Materials, Beijing University of Chemical Technology, Beijing, 100029, China
[4]State Key Laboratory of Organic-Inorganic Composites, Beijing University of Chemical Technology, Beijing 100029, China
[5]These authors contributed equally: Zhuangzhuang Qiao, Kai Zhang.

* To whom correspondence should be addressed
Email: xufj@mail.buct.edu.cn, zhaonn@mail.buct.edu.cn

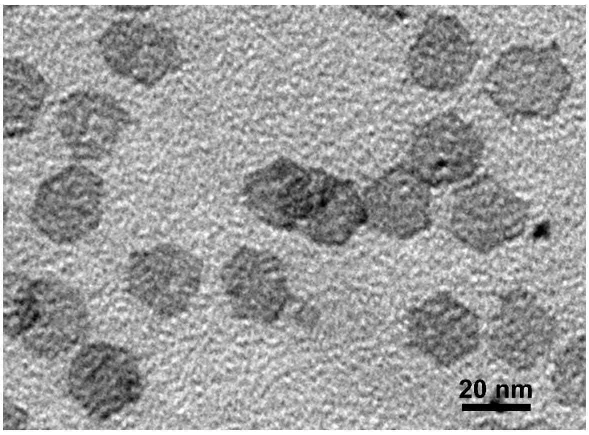

Supplementary Fig. 1. TEM characterization. TEM image of Pd nanosheets. Experiments were repeated three times independently with similar results.

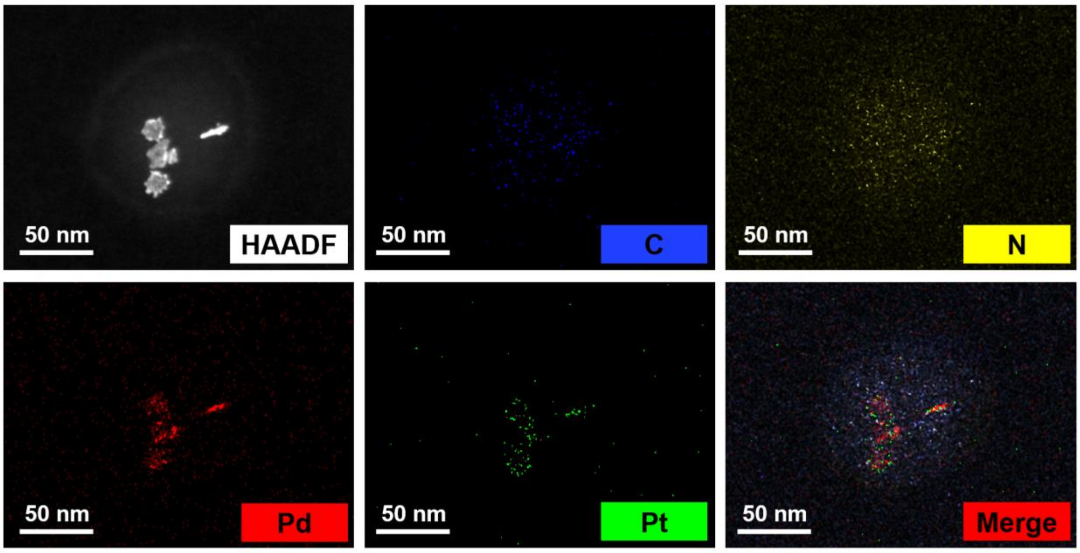

Supplementary Fig. 2. Elemental characterization. Elemental mapping of EV-Pd-Pt nanoparticles. Experiments were repeated three times independently with similar results.

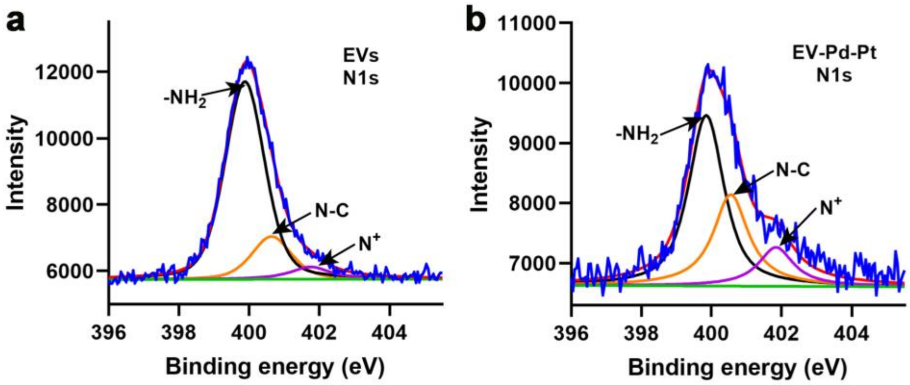

Supplementary Fig. 3. Characterization of chemical bonding by XPS. High-resolution X-ray photoelectron spectroscopy spectra of N1s in (**a**) EVs and (**b**) EV-Pd-Pt. Source data are provided as a Source Data file.

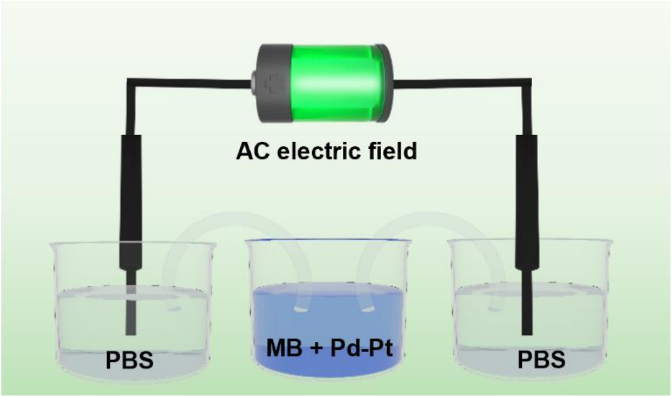

Supplementary Fig. 4. Schematic diagram of the double salt bridge system. Schematic illustration of the equipment for electro-driven catalytic experiment.

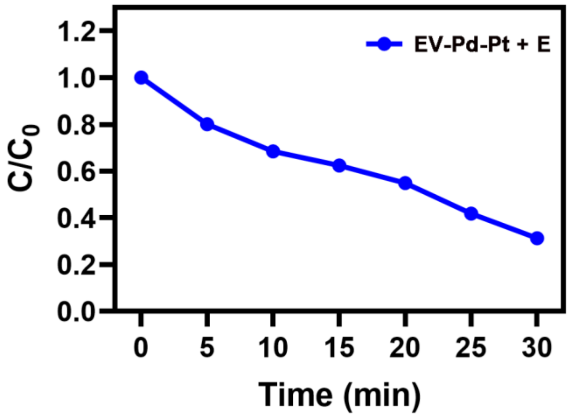

Supplementary Fig. 5. Characterization of MB degradation. Degradation rate of MB in the presence of EV-Pd-Pt (Pd-Pt concentration: 50 μg/mL) under electric field. Source data are provided as a Source Data file.

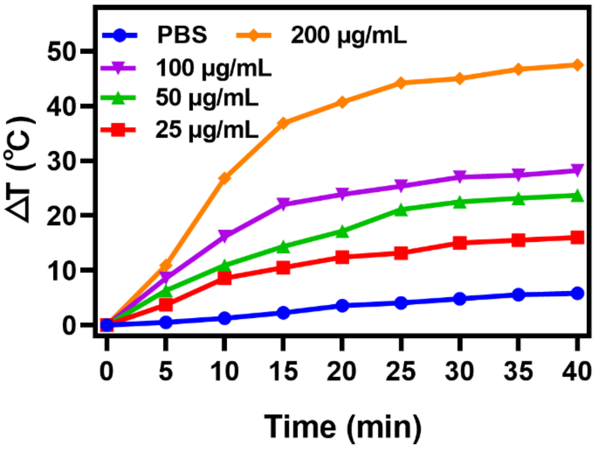

Supplementary Fig. 6. Photothermal property of Pd-Pt nanosheets. Temperature evolution curves of Pd-Pt solutions with different concentrations under 980 nm laser irradiation (0.5 W/cm). Source data are provided as a Source Data file.

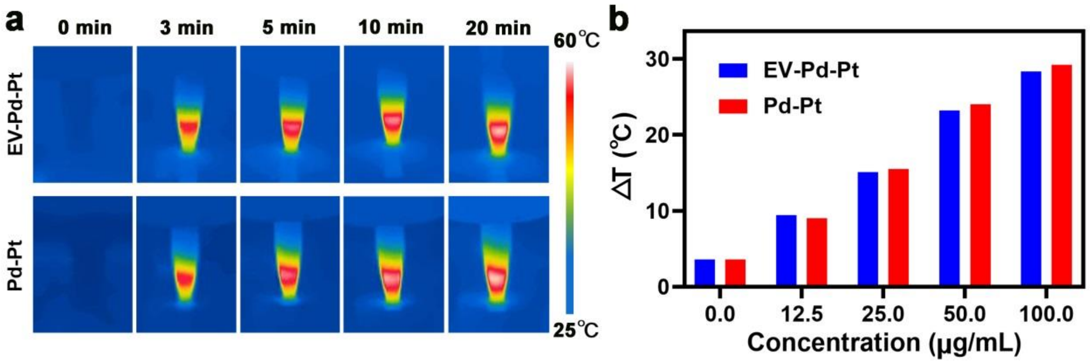

Supplementary Fig. 7. Photothermal property of EV-Pd-Pt. a, Infrared thermal images of EV-Pd-Pt and Pd-Pt (Pd-Pt concentration: 100 μg/mL) under 980 nm laser irradiation (0.5 W/cm^2) for 20 min. **b**, Temperature variations of EV-Pd-Pt and Pd-Pt solutions at different Pd-Pt concentrations under 980 nm laser irradiation. Source data are provided as a Source Data file.

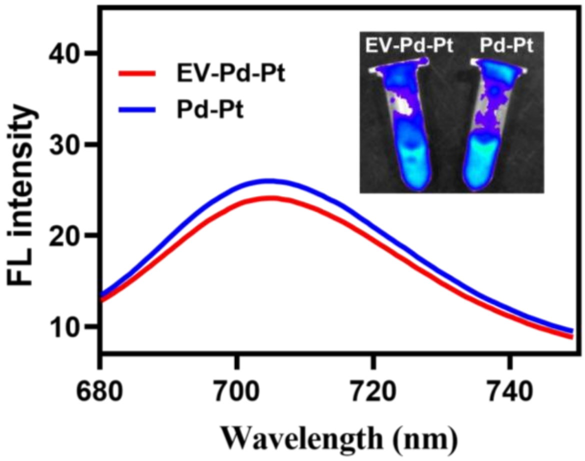

Supplementary Fig. 8. Characterization of fluorescently labeled nanoparticles. Fluorescence emission spectra of Cy5.5-labeled Pd-Pt and EV-Pd-Pt with excitation at 640 nm. Source data are provided as a Source Data file.

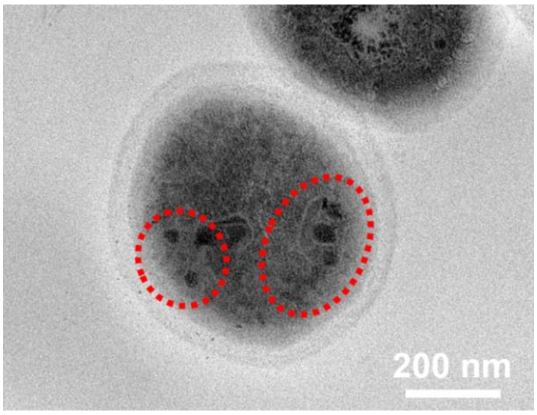

Supplementary Fig. 9. TEM characterization of the bacterial section. TEM images of an ultrathin section of *S. aureus* incubated with EV-Pd-Pt for 0.5 h. Experiments were repeated three times independently with similar results.

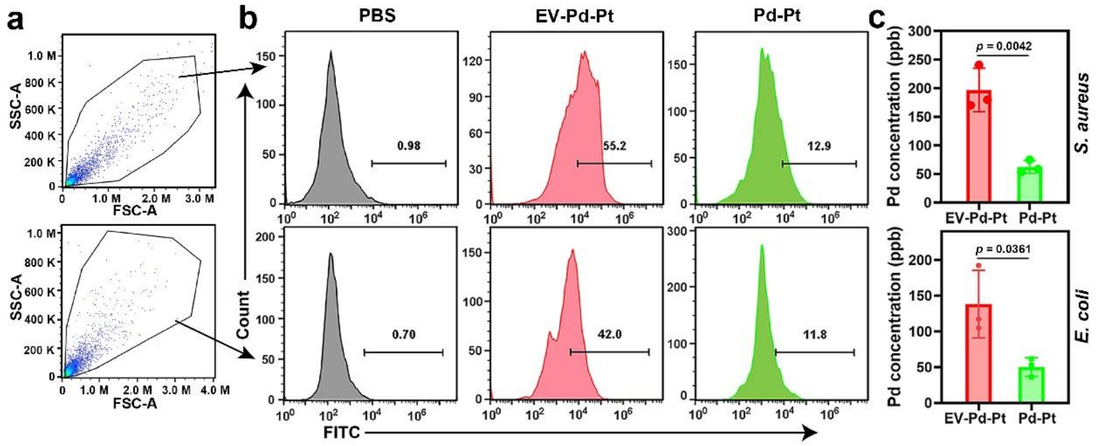

Supplementary Fig. 10. Uptake of different nanoparticles by bacteria analyzed by flow cytometry and ICP-MS. The gating strategy (**a**) and the flow cytometric analysis (**b**) of bacteria incubated with EV-Pd-Pt and Pd-Pt labeled by fluorescein-5-isothiocyanate (FITC) for 30 min, respectively. **c**, Quantification analysis of EV-Pd-Pt and Pd-Pt taken up by *S. aureus* and *E. coli* determined by ICP-MS. Data are presented as mean values ± SD (n = 3 independent samples). Statistical significance was calculated by two-tailed Student's t-test. Source data are provided as a Source Data file.

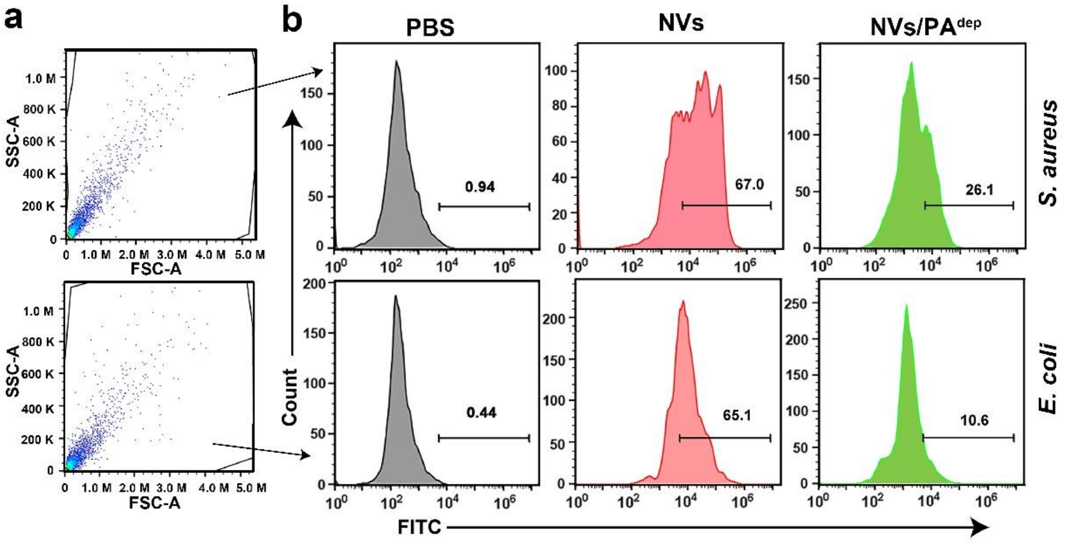

Supplementary Fig. 11. Uptake of EV-Pd-Pt by different nano-sized vesicles analyzed by flow cytometry. The gating strategy (**a**) and the flow cytometric analysis (**b**) of bacteria incubated with NVs assembled from total lipids and PA-depleted lipids, respectively.

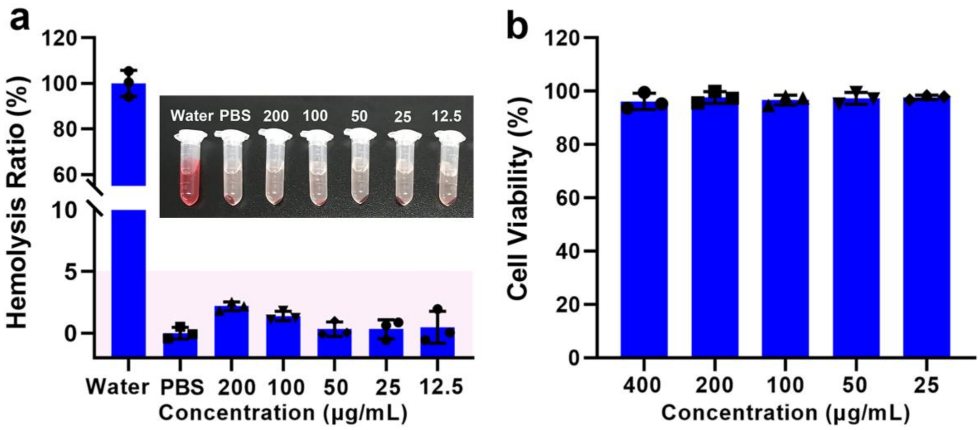

Supplementary Fig. 12. Biocompatibility of EV-Pd-Pt nanoparticles in vitro. a, Relative hemolysis ratio of EV-Pd-Pt solutions with different concentrations. **b**, Cell viability of L929 cells after 24 h incubation with EV-Pd-Pt solutions with different concentrations. Data are presented as mean values ± SD (*n* = 3 independent samples). Source data are provided as a Source Data file.

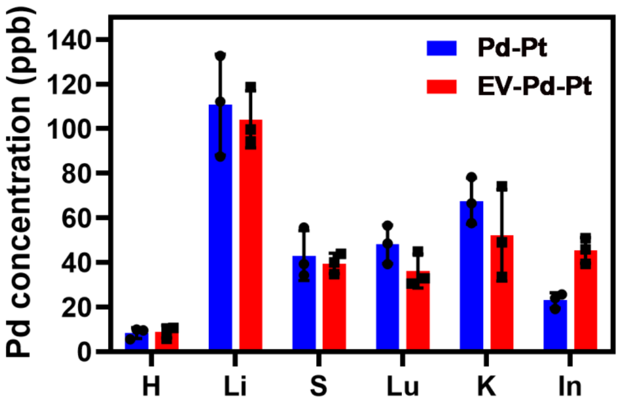

Supplementary Fig. 13. Quantitative analysis of nanoparticles in major organs. Biodistribution of EV-Pd-Pt and Pd-Pt evaluated by Pd in different organs at 8 h post-injection determined by ICP-MS. Data are presented as mean values ± SD (*n* = 3 independent samples). Source data are provided as a Source Data file.

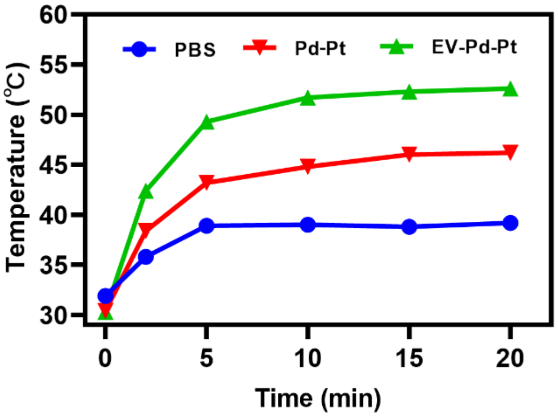

Supplementary Fig. 14. Photothermal effect of nanoparticles in vivo. Corresponding temperature profiles of infection sites after intravenous injection with PBS, Pd-Pt, and EV-Pd-Pt, respectively as a function of irradiation time (980 nm, 0.5 W/cm^2). Source data are provided as a Source Data file.

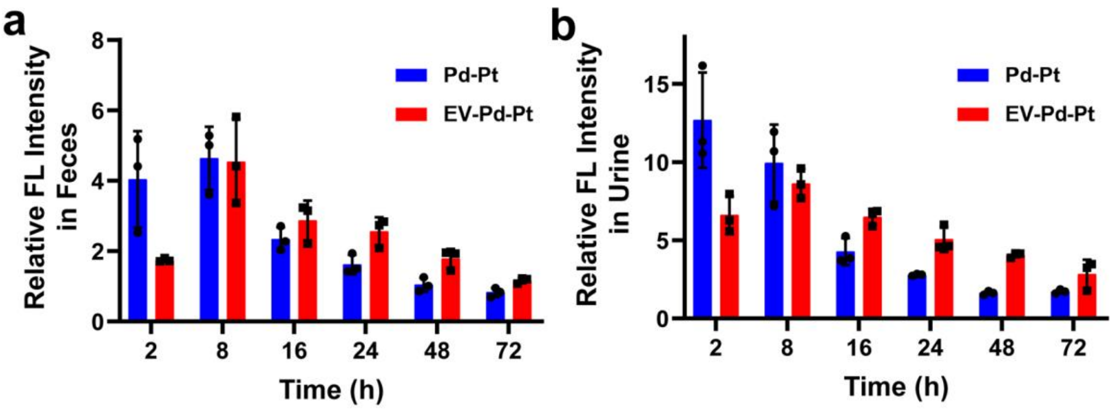

Supplementary Fig. 15. Evaluation of in vivo excretion by fluorescence signals. Quantitative analysis the fluorescence intensity of feces (**a**) and urine (**b**) at different time points after intravenous injection of Pd-Pt or EV-Pd-Pt. Data are presented as mean values ± SD (*n* = 3 independent samples). Source data are provided as a Source Data file.

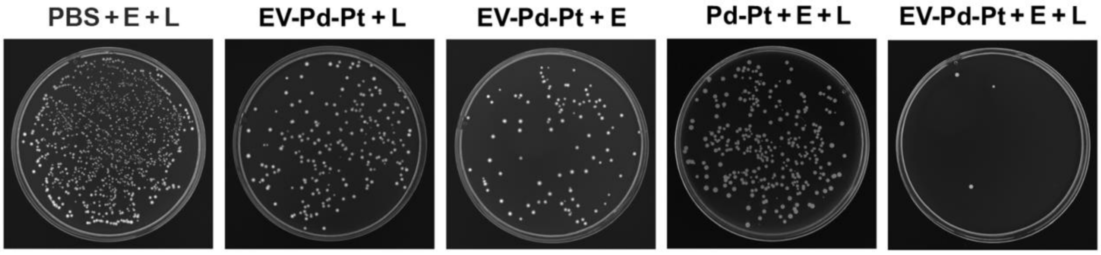

Supplementary Fig. 16. In vivo antibacterial effect evaluated by plate counting method. Photographs of bacterial colonies from infected tissues of different treatment groups after 5 days of treatment.

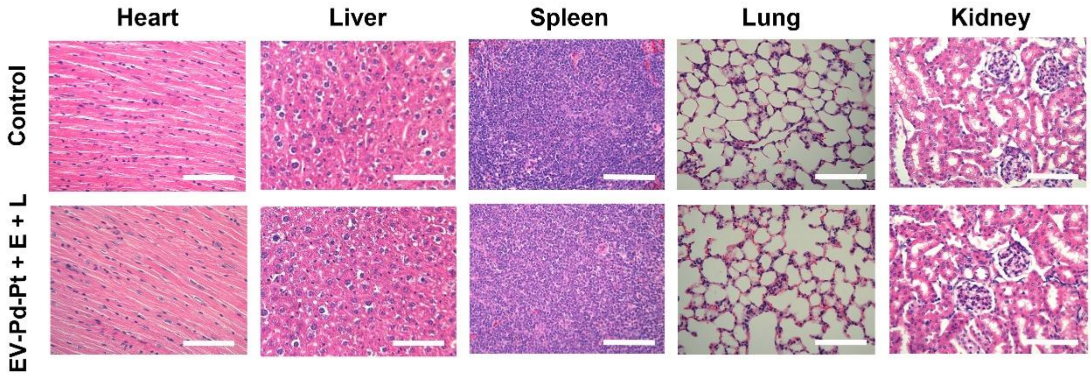

Supplementary Fig. 17. In vivo biocompatibility evaluated by histological analysis. Corresponding H&E staining of major organs including heart, liver, spleen, lungs, and kidneys in EV-Pd-Pt + E + L group after 5 days of treatment. Healthy mice served as controls. Scale bar: 100 μm. Experiments were repeated three times independently with similar results.

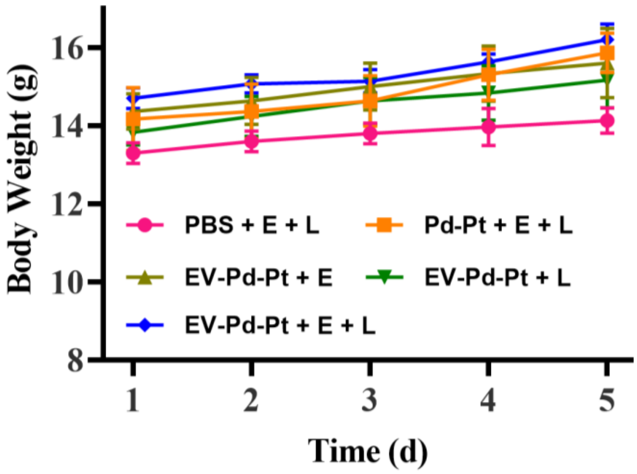

Supplementary Fig. 18. Evaluation of changes in mouse body weight. Body weight changes of mice in different groups after the treatments. Data are presented as mean values ± SD (n = 3 independent samples). Source data are provided as a Source Data file.

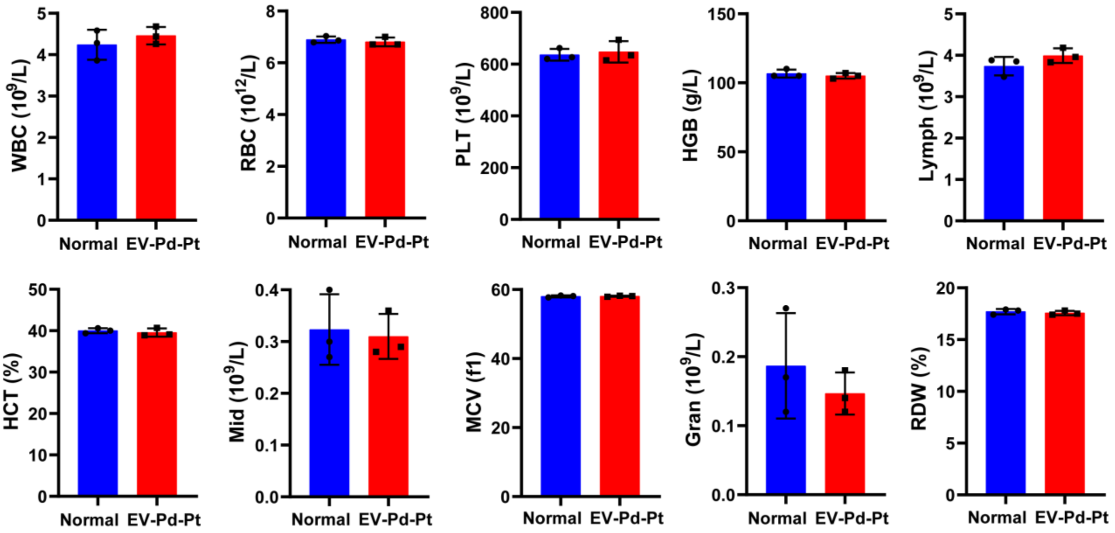

Supplementary Fig. 19. Blood biochemical analysis. Some blood routine and biochemical indexes of mice in EV-Pd-Pt + E + L group after 5 days of treatment. Data are presented as mean values ± SD (n = 3 independent samples). Source data are provided as a Source Data file.
