# Supplementary material for: Biomimetic electrodynamic nanoparticles comprising ginger-derived extracellular vesicles for synergistic anti-infective therapy
Source: Nat Commun. 2022 Nov 22;13:7164. doi: 10.1038/s41467-022-34883-5 (PMC9684156; doi:10.1038/s41467-022-34883-5)
Supplement: Supplementary file 2 — Reporting Summary [file 41467_2022_34883_MOESM2_ESM.pdf]

nature portfolio

Corresponding author(s): Nana Zhao and Fu-Jian Xu

Last updated by author(s): Nov 7, 2022

Reporting Summary

Nature Portfolio wishes to improve the reproducibility of the work that we publish. This form provides structure for consistency and transparency in reporting. For further information on Nature Portfolio policies, see our Editorial Policies and the Editorial Policy Checklist.

Statistics

For all statistical analyses, confirm that the following items are present in the figure legend, table legend, main text, or Methods section.

n/a	Confirmed	
[ ]	[x]	The exact sample size (*n*) for each experimental group/condition, given as a discrete number and unit of measurement
[ ]	[x]	A statement on whether measurements were taken from distinct samples or whether the same sample was measured repeatedly
[ ]	[x]	The statistical test(s) used AND whether they are one- or two-sided<br>*Only common tests should be described solely by name; describe more complex techniques in the Methods section.*
[x]	[ ]	A description of all covariates tested
[x]	[ ]	A description of any assumptions or corrections, such as tests of normality and adjustment for multiple comparisons
[ ]	[x]	A full description of the statistical parameters including central tendency (e.g. means) or other basic estimates (e.g. regression coefficient) AND variation (e.g. standard deviation) or associated estimates of uncertainty (e.g. confidence intervals)
[ ]	[x]	For null hypothesis testing, the test statistic (e.g. *F*, *t*, *r*) with confidence intervals, effect sizes, degrees of freedom and *P* value noted<br>*Give P values as exact values whenever suitable.*
[x]	[ ]	For Bayesian analysis, information on the choice of priors and Markov chain Monte Carlo settings
[x]	[ ]	For hierarchical and complex designs, identification of the appropriate level for tests and full reporting of outcomes
[x]	[ ]	Estimates of effect sizes (e.g. Cohen's *d*, Pearson's *r*), indicating how they were calculated

Our web collection on statistics for biologists contains articles on many of the points above.

Software and code

Policy information about availability of computer code

Data collection	Vienna ab Initio Simulation Package 5.4.1 Code, Leica Application Suite Advanced Fluorescence Lite (LAS AF Lite) 3.3.0.10134, BD CSampler Plus software 1.0.27.1, Living Image 4.7.3, PA Imaging System 3.5.0.
Data analysis	Microsoft Office Excel 2019, GraphPad Prism (version 8.0.2), FlowJo_V10 software (version 10.7.1)

For manuscripts utilizing custom algorithms or software that are central to the research but not yet described in published literature, software must be made available to editors and reviewers. We strongly encourage code deposition in a community repository (e.g. GitHub). See the Nature Portfolio guidelines for submitting code & software for further information.

Data

Policy information about availability of data

All manuscripts must include a data availability statement. This statement should provide the following information, where applicable:

- Accession codes, unique identifiers, or web links for publicly available datasets
- A description of any restrictions on data availability
- For clinical datasets or third party data, please ensure that the statement adheres to our policy

Data supporting the findings of this study are available within the article and the Supplementary Information. Source data are available for Figs. 2e, h–k, 3a-c, e, 5c, 6a, b, 7c, e, g, j, 8c-d and Supplementary Figs. 3, 5, 6, 7b, 8, 10b, 12-15, 18 and 19 in the associated source data file. Other data are available from the corresponding authors upon reasonable request.

Human research participants

Policy information about studies involving human research participants and Sex and Gender in Research.

Reporting on sex and gender	Not applicable
Population characteristics	Not applicable
Recruitment	Not applicable
Ethics oversight	Not applicable

Note that full information on the approval of the study protocol must also be provided in the manuscript.

Field-specific reporting

Please select the one below that is the best fit for your research. If you are not sure, read the appropriate sections before making your selection.

☒ Life sciences ☐ Behavioural & social sciences ☐ Ecological, evolutionary & environmental sciences

For a reference copy of the document with all sections, see nature.com/documents/nr-reporting-summary-flat.pdf

Life sciences study design

All studies must disclose on these points even when the disclosure is negative.

Sample size	Sample sizes were predetermined from pilot experiments and/or experiments that have been done in the past, to obtain statistically significant data.
Data exclusions	No data were excluded from analysis.
Replication	All experiments were performed with independent replicates as described in the figure legends.
Randomization	All samples were randomly allocated into experimental groups.
Blinding	Investigators were blinded to group allocation during data collection and analysis for both in vitro and in vivo experiments.

Reporting for specific materials, systems and methods

We require information from authors about some types of materials, experimental systems and methods used in many studies. Here, indicate whether each material, system or method listed is relevant to your study. If you are not sure if a list item applies to your research, read the appropriate section before selecting a response.

Materials & experimental systems

n/a	Involved in the study	
☒	☐	Antibodies
☐	☒	Eukaryotic cell lines
☒	☐	Palaeontology and archaeology
☐	☒	Animals and other organisms
☒	☐	Clinical data
☒	☐	Dual use research of concern

Methods

n/a	Involved in the study	
☒	☐	ChIP-seq
☐	☒	Flow cytometry
☒	☐	MRI-based neuroimaging

Eukaryotic cell lines

Policy information about cell lines and Sex and Gender in Research

Cell line source(s)	L 929 cells were obtained the American Type Culture Collection (ATCC).
Authentication	No further authentication was done after the cells were obtained from the vendors.
Mycoplasma contamination	All cell lines were tested for mycoplasma contamination. No mycoplasma contamination was found.
Commonly misidentified lines (See ICLAC register)	No commonly misidentified cell lines were used.

Animals and other research organisms

Policy information about studies involving animals; ARRIVE guidelines recommended for reporting animal research, and Sex and Gender in Research

Laboratory animals	BALB/c mice (female, 4-6 weeks old) were purchased from Vital River Laboratory Animal Technology Co. Ltd (Beijing, China) and housed with a 12 h light/dark cycle. The ambient temperature was 22 degree Celsius and humidity was 30-70 %.
Wild animals	The study did not involve wild animals.
Reporting on sex	The findings apply to both sexes, and gender was not considered in the study design.
Field-collected samples	The study did not involve samples collected from the field.
Ethics oversight	Animal studies were approved by Ethical Committee of Chinese Academy of Medical Sciences and Peking Union Medical College and performed under legal protocols.

Note that full information on the approval of the study protocol must also be provided in the manuscript.

Flow Cytometry

Plots

Confirm that:

- [x] The axis labels state the marker and fluorochrome used (e.g. CD4-FITC).
- [x] The axis scales are clearly visible. Include numbers along axes only for bottom left plot of group (a 'group' is an analysis of identical markers).
- [ ] All plots are contour plots with outliers or pseudocolor plots.
- [x] A numerical value for number of cells or percentage (with statistics) is provided.

Methodology

Sample preparation	Cells were mixed with fluorescence-labelled samples following the manufacturer's instructions.
Instrument	BD Accuri C6 plus (BD Biosciences)
Software	BD CSampler Plus software (version 1.0.27.1) and FlowJo_V10 software (version 10.7.1)
Cell population abundance	The instrument counts 10,000 cells autonomously
Gating strategy	Cells was gated on FSC/SSC.

- [x] Tick this box to confirm that a figure exemplifying the gating strategy is provided in the Supplementary Information.
